# Alternative Splicing Events Are a Late Feature of Pathology in a Mouse Model of Spinal Muscular Atrophy

**DOI:** 10.1371/journal.pgen.1000773

**Published:** 2009-12-18

**Authors:** Dirk Bäumer, Sheena Lee, George Nicholson, Joanna L. Davies, Nicholas J. Parkinson, Lyndsay M. Murray, Thomas H. Gillingwater, Olaf Ansorge, Kay E. Davies, Kevin Talbot

**Affiliations:** 1 MRC Functional Genomics Unit, Department of Physiology, Anatomy and Genetics, University of Oxford, Oxford, United Kingdom; 2 Department of Statistics, University of Oxford, Oxford, United Kingdom; 3 Centre for Integrative Physiology and Euan MacDonald Centre for Motor Neuron Disease Research, University of Edinburgh Medical School, Edinburgh, United Kingdom; 4 Department of Neuropathology, John Radcliffe Hospital, Oxford, United Kingdom; 5 Department of Clinical Neurology, University of Oxford, John Radcliffe Hospital, Oxford, United Kingdom; University of Washington, United States of America

## Abstract

Spinal muscular atrophy is a severe motor neuron disease caused by inactivating mutations in the *SMN1* gene leading to reduced levels of full-length functional SMN protein. SMN is a critical mediator of spliceosomal protein assembly, and complete loss or drastic reduction in protein leads to loss of cell viability. However, the reason for selective motor neuron degeneration when SMN is reduced to levels which are tolerated by all other cell types is not currently understood. Widespread splicing abnormalities have recently been reported at end-stage in a mouse model of SMA, leading to the proposition that disruption of efficient splicing is the primary mechanism of motor neuron death. However, it remains unclear whether splicing abnormalities are present during early stages of the disease, which would be a requirement for a direct role in disease pathogenesis. We performed exon-array analysis of RNA from SMN deficient mouse spinal cord at 3 time points, pre-symptomatic (P1), early symptomatic (P7), and late-symptomatic (P13). Compared to littermate control mice, SMA mice showed a time-dependent increase in the number of exons showing differential expression, with minimal differences between genotypes at P1 and P7, but substantial variation in late-symptomatic (P13) mice. Gene ontology analysis revealed differences in pathways associated with neuronal development as well as cellular injury. Validation of selected targets by RT–PCR confirmed the array findings and was in keeping with a shift between physiologically occurring mRNA isoforms. We conclude that the majority of splicing changes occur late in SMA and may represent a secondary effect of cell injury, though we cannot rule out significant early changes in a small number of transcripts crucial to motor neuron survival.

## Author Summary

The identification of mutations in the Survival Motor Neuron (SMN) gene as the cause of the severe motor neuron disorder spinal muscular atrophy is one of a number of discoveries implicating selective motor neuron vulnerability to defects in processing of RNA and its associated ribonucleoprotein complexes. An unresolved issue is whether loss of the general cellular function of SMN in spliceosomal assembly, which is predicted to result in widespread defects in mRNA splicing, is directly responsible for motor neuron death. We have used exon-specific microarrays to assess the degree of altered splicing in the spinal cord in a mouse model of SMA. Our finding that the vast majority of splicing changes are a late feature of the disease and may represent a shift to alternative isoform expression, rather than loss of splicing fidelity, provides evidence that widespread splicing disturbance is not a primary feature of the disease pathogenesis but a secondary effect of cell injury in a late phase of the disease. However, our study cannot rule out a role for subtle early changes in one or a few transcripts crucial to motor neuron survival expressed at low levels or in only in a sub-population of spinal cord cells.

## Introduction

Autosomal recessive Spinal Muscular Atrophy (SMA) is a leading genetic cause of infant mortality, with a carrier frequency of 1∶50 and an annual incidence of 1 in 10,000 live births [1]. Affected individuals develop symmetrical, proximal weakness resulting from neurogenic muscle atrophy, and ultimately leading, in the most severely affected individuals, to death from respiratory failure. The pathological correlate of these symptoms is selective loss of large alpha motor neurons in the ventral horn of the spinal cord. The vast majority of cases are caused by homozygous deletion of the survival motor neuron 1 (*SMN1*) gene [2] with subsequent reduction in levels of the SMN protein [3]. SMN is highly conserved in evolution and ubiquitously expressed. Complete loss of SMN, which is incompatible with life [4], is prevented by production of SMN from the *SMN2* gene, a near identical paralogue of *SMN1* which has arisen from an inverted duplication event in recent evolution. The presence of a translationally silent C-T transition in *SMN2* exon 7, however, leads to disruption of an exonic splice-enhancer (ESE) element and exon 7 skipping in the majority of *SMN2* derived transcripts [5],[6]. The functional consequence is that *SMN2* produces only very little full length SMN (FL-SMN), while the SMND7 isoform is translated into an unstable protein that is rapidly degraded [7]. Disease severity is broadly proportional to residual SMN levels, which is a function of *SMN2* copy number, although other modifying factors are involved in some cases [8],[9].

The mechanism by which SMN deficiency leads to selective lower motor neuron loss in SMA is poorly understood and difficult to reconcile with its ubiquitous expression unless either SMN has a motor neuron-specific function or motor neurons are selectively vulnerable to a deficiency in the general function of SMN common to all cells. Currently, the best characterised function of SMN is as part of a multi-protein complex which is critical for the core steps in the assembly of small nuclear ribonuclear proteins (snRNPs), components of the spliceosome, the cellular machinery that controls splicing of pre-mRNAs [10],[11]. In particular, SMN acts in the cytoplasmic assembly of Sm core proteins on snRNAs, which is a prerequisite for import of snRNPs into the nucleus [12]. SMN levels and the activity of snRNP assembly vary during development and according to tissue type. In the mouse spinal cord, snRNP assembly is highest during embryogenesis and early postnatal development and then falls to a baseline level when myelination occurs [13]. Mouse models of SMA, which have reduced SMN levels, show a drop in snRNP assembly activity as measured by *in vitro* assays, while steady state snRNP levels measured in tissues are only mildly reduced [14]. Interestingly, a subset of snRNPs belonging to the minor spliceosome seems to be differentially affected [14],[15]. Several strands of evidence support the notion that reduced snRNP assembly is associated with motor neuron degeneration. The subtle motor neuron loss that has been reported by some investigators at late stages in mice heterozygous null for *Smn* can be accelerated by crossing with mice heterozygous null for Gemin2, another core component of the SMN complex. This is associated with a reduction in snRNP assembly [16]. In zebrafish, a failure of embryonic motor axon growth can be induced by silencing not only SMN, but also gemin2. The observed defects can be rescued by direct injection of U snRNPs [17]. However, other studies in zebrafish indicate that the axonal degeneration phenotype is not coupled to U snRNP assembly [18]. Reducing SMN levels in HeLa cells by RNAi leads to an increased error rate in splice-site pairing, which has also been observed in fibroblasts from SMA patients [19]. Finally, a recent study in SMA mice found that at end-stage, widespread splicing abnormalities can be found in several tissues including the spinal cord. Importantly, different transcripts were found to be altered in a tissue-specific manner [15].

While both snRNP assembly dysfunction and splicing abnormalities have been documented in models of SMA, several questions remain. It is still unclear whether splicing abnormalities cause motor neuron loss in SMA, or whether they are a late occurrence in disease, either as a consequence of spliceosome dysfunction or the severe physiological alterations secondary to respiratory distress, hypoxia and malnutrition. If spliceosome dysfunction is critical for disease pathogenesis, the mechanism of splicing alterations needs to be further delineated. In addition, the role of SMN in spliceosome assembly is only one of many functions that are potentially altered in SMA, including roles in transcription regulation [20]–[22], axonal transport of mRNA and RNPs [23],[24] as well as regulation of local translation at the neuromuscular junction [25].

This study had two aims. First, we wanted to address the question of whether abnormal splicing events are a cause or consequence of SMA. We utilised an exon-specific microarray to examine the transcriptome of spinal cord samples harvested from SMA and control mice at pre-symptomatic, early symptomatic and late-symptomatic stages, to test the hypothesis that widespread alteration of splicing precedes disease onset. Secondly, we used the difference in temporal mRNA expression pattern between SMA and control mice to identify neuronal pathways disturbed by SMN deficiency.

## Results

### Correlation of mouse phenotype and motor neuron loss

**Figure 1:**
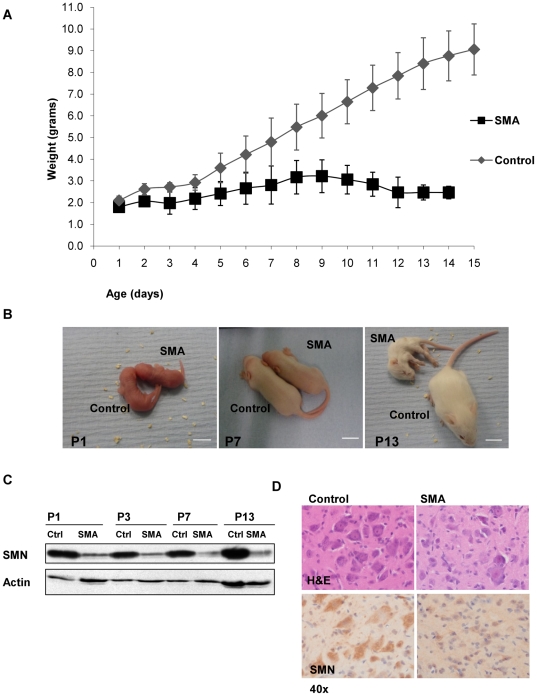
Phenotype of the SMNΔ7mouse model. (A) Post-natal weight development of SMA (Smn−/−;SMN2;SMNΔ7) and control (Smn+/+;SMN2;SMNΔ7) mice. Average weight of 4 animals per genotype and time-point; error-bars represent standard deviation of the mean. (B) Representative images of SMA and control mice littermates at P1, P7, and P13. Scale bar 1 cm. Genotypes can be reliably distinguished morphologically from P7 onwards. (C) Western blot of whole spinal cord lysates show markedly reduced SMN levels at all measured time points in the SMA mice. (D) At P13, paucity of large motor neurons in the ventral horn is apparent on H&E stain, with reduced SMN immunoreactivity on immunohistochemistry.

**Figure 2:**
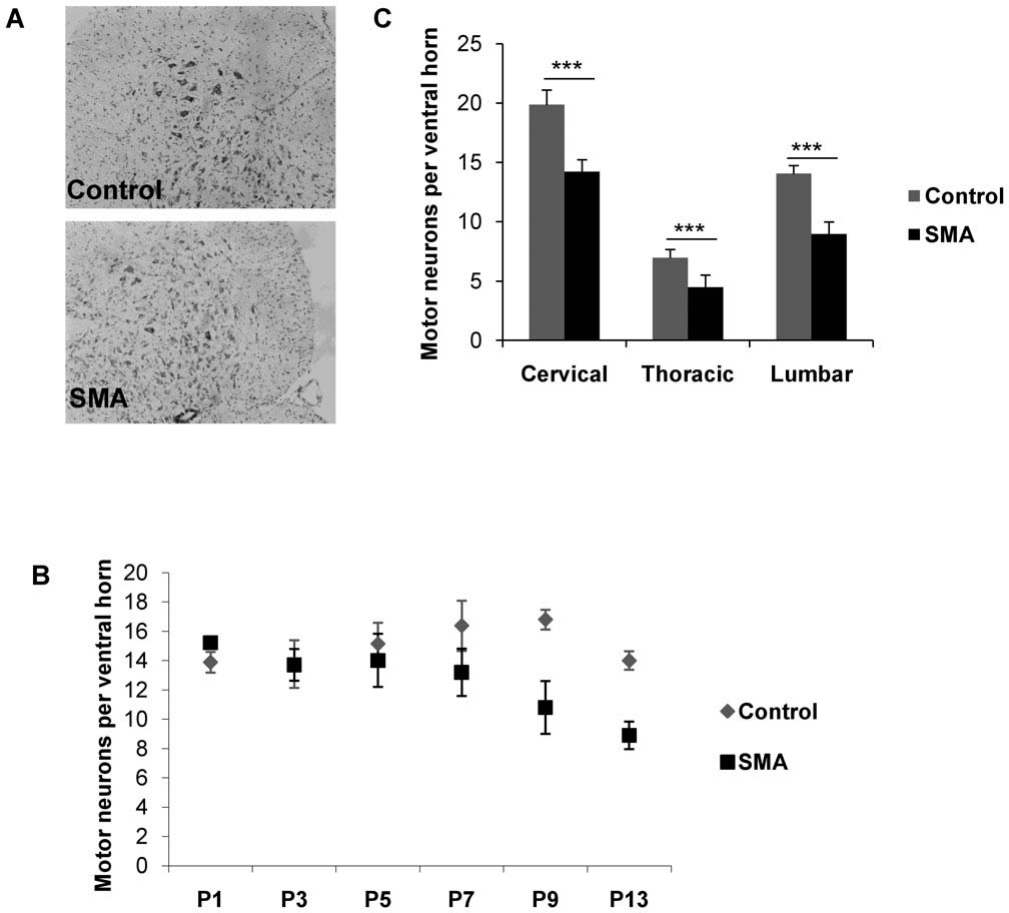
Motor neuron loss in SMA mice. (A) Cresyl violet stain of spinal cord sections showing the ventral horn in control (top) and SMA mice (bottom), with relative reduction of large Nissl dense cells in SMA mice. (B) Lumbar motor neurons were counted in five animals per genotype at post-natal days P1, P3, P5, P7, P9, and P13. Motor neuron numbers were equal at the pre-symptomatic time points, while motor neuron loss became detectable at P7 followed by further decline to approximately 65% of control animal numbers at late-symptomatic stage. Error bars represent the standard deviation of the mean. (C) At P13, motor neuron loss affects all spinal cord segments, although the absolute number of motor neurons is higher in the cervical and lumbar region, reflecting the innervation of forelimbs and hind limbs.

The mouse model used in this study (*Smn−/−; SMN2; SMN*Δ*7*) is the most commonly used model of severe SMA and has been described previously [26]. The maximum lifespan of SMA mice was 14 days. Subtle differences in weight compared to the control littermates (*Smn+/+; SMN2; SMN*Δ*7*) were discernible before P7, but were not reliably predictive of genotype in individual mice (Figure 1A and 1B). SMN protein levels were markedly reduced in SMA mice at all time points as measured by Western blot (Figure 1C) and immunohistochemistry (Figure 1D). At P7 a failure in the righting reflex became apparent. Importantly, this coincided with a drop in numbers of large motor neurons in the spinal cord (Figure 2A and 2B). No discernible differences in phenotype or pathology were present at P1, indicating that, in the SMNΔ7 mouse model of SMA, the disease develops in a motor system in which embryonic development has been relatively normal. By P13, mice appeared emaciated, were unable to right themselves, and showed signs of respiratory distress (Figure 1B). There was a corresponding loss of >30% of lower motor neurons from the ventral horn in SMA mice. At late-symptomatic stage (P13), a similar relative loss of motor neurons was evident across the entire length of the spinal cord, (Figure 2C), justifying the use of whole spinal cord for RNA analysis. However, absolute numbers differed depending on the region examined, reflecting the differential innervation of limb and trunk musculature by motor neurons originating in the cervical, thoracic or lumbar cord.

### Global changes in the spinal cord transcriptome

**Figure 3:**
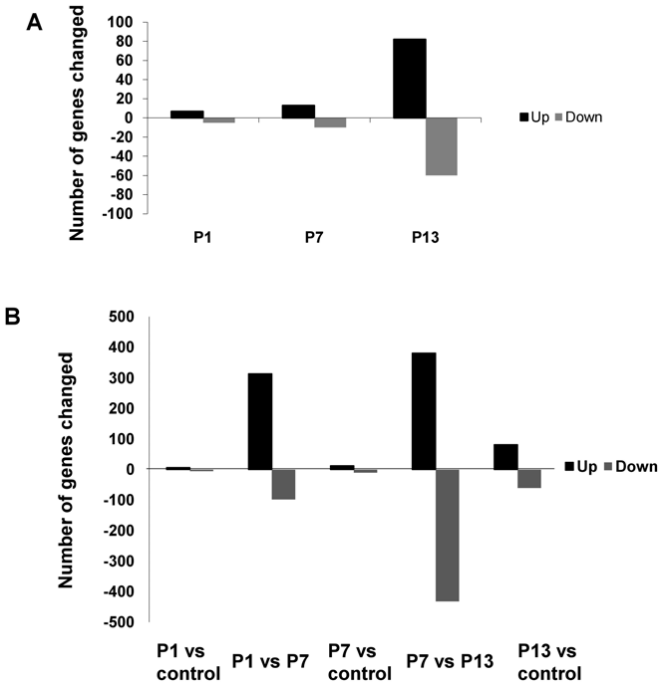
Global transcriptome changes increase over time. (A) Number of genes up- or down- regulated more than 1.5 fold in SMA mice compared to control using a p-value threshold of ≤0.05. There is a major increase in gene expression change at late-symptomatic compared to pre-symptomatic and early-symptomatic stages. (B) Gene expression changes in control mice (*Smn+/+*) between time points are of much larger scale than changes between genotypes.

To understand the scale of change in mRNA expression in the spinal cord, we first performed gene-level comparisons between genotypes at each time point, using the core probe sets on the array and GeneSpring software (Agilent Technologies, Santa Clara, CA, USA). When using an arbitrary p-value threshold of 0.05 and a fold-change threshold of 1.5 as cut-off for biological significance, the expression of 142 genes was increased or decreased in the spinal cord of SMA mice compared with their control littermates at late-symptomatic stage (P13), with a maximal fold-change of 3.9 (Table S1). Importantly, the degree of change between SMA and control mice was much smaller at the pre-symptomatic (P1) and early-symptomatic (P7) stages, with only 12 and 23 genes changed, respectively (Table 1 and Table 2; Figure 3A). This finding argues against a critical function of SMN in transcription regulation, but also shows that if widespread splicing changes occur in SMA, this does not lead to a pre-symptomatic systemic change in whole transcript level mRNA expression, which might be expected if mis-spliced transcripts are subject to nonsense-mediated decay.

**Table 1:** P7 gene-level changes SMA versus control, fold change >1.5, P δ 0.05.

Transcripts Cluster Id	Fold change	Regulation	Gene Title	Gene Symbol
6809524	3.5	down	survival motor neuron 1	Smn1
6969997	2.6	down	hemoglobin, beta adult minor chain///hemoglobin, beta adult major chain	Hbb-b2///Hbb-b1
6805381	2.2	up	histone cluster 1, H1c	Hist1h1c
6849595	2.2	up	cyclin-dependent kinase inhibitor 1A (P21)	Cdkn1a
7018366	2.1	up	ectodysplasin A2 isoform receptor	Eda2r
6961201	1.9	up	small nuclear ribonucleoprotein polypeptide A′	Snrpa1
6899760	1.9	up	thioredoxin interacting protein	Txnip
6755306	1.7	down	olfactory receptor 420	Olfr420
6842587	1.7	down	Chondrolectin	Chodl
6790294	1.7	up	chemokine (C-C motif) ligand 3	Ccl3
6764650	1.6	up	epoxide hydrolase 1, microsomal	Ephx1
6754143	1.6	up	ribonuclease L (2′, 5′-oligoisoadenylate synthetase-dependent)	Rnasel
6926165	1.6	up	complement component 1, q subcomponent, beta polypeptide	C1qb
6963128	1.6	up	olfactory receptor 635	Olfr635
6873271	1.6	down	stearoyl-Coenzyme A desaturase 1	Scd1
6785114	1.6	down	RAB37, member of RAS oncogene family	Rab37
6926166	1.6	up	complement component 1, q subcomponent, C chain	C1qc
7009748	1.6	down	diacylglycerol kinase kappa	Dgkk
6788617	1.6	down	olfactory receptor 323	Olfr323
6885616	1.5	up	RIKEN cDNA 1700007K13 gene	1700007K13Rik
6939671	1.5	down	transmembrane protease, serine 11d	Tmprss11d
6870375	1.5	up	insulin I	Ins1
6783144	1.5	down	carbonic anhydrase 4	Car4

**Table 2:** P1 gene-level changes SMA versus control, fold change >1.5, P δ 0.05.

Transcripts Cluster Id	Fold change	Regulation	Gene Title	Gene Symbol
6809524	3.4	down	survival motor neuron 1	Smn1
6805381	1.8	up	histone cluster 1, H1c	Hist1h1c
6805370	1.8	up	histone cluster 1, H2bc///histone cluster 1, H2bj///histone cluster 1, H2bk///histone cluster 1, H2bf///histone cluster 1, H2bl///histone cluster 1, H2bn///histone cluster 1, H2bb///histone cluster 1, H2be///histone cluster 1, H2bg///predicted gene, OTTMUSG00000013203	Hist1h2bc///Hist1h2bj///Hist1h2bk///Hist1h2bf///Hist1h2bl///Hist1h2bn///Hist1h2bb///Hist1h2be///Hist1h2bg///RP23-38E20.1
6767782	1.8	up	glycoprotein 49 A///leukocyte immunoglobulin-like receptor, subfamily B, member 4	Gp49a///Lilrb4
6780730	1.6	down	olfactory receptor 1393///olfactory receptor 1392	Olfr1393///Olfr1392
6772906	1.6	up	laminin, alpha 2	Lama2
6988353	1.6	down	olfactory receptor 978	Olfr978
6960235	1.6	up	kallikrein 1-related peptidase b21///kallikrein 1-related peptidase b24///kallikrein 1-related peptidase b11///kallikrein 1-related peptidase b27	Klk1b21///Klk1b24///Klk1b11///Klk1b27
6973490	1.6	down	---	---
6839959	1.6	down	polymerase (RNA) II (DNA directed) polypeptide H	Polr2h
6992950	1.5	down	ribosomal protein L14///RIKEN cDNA 5830454E08 gene	Rpl14///5830454E08Rik
6963442	1.5	down	Adrenomedullin	Adm

An additional data analysis, which we refer to as the ENSG analsysis, was performed in which probes were grouped into sets, each corresponding to a gene in the Ensembl (version 49) annotation database [27]. Time point-specific differential expression between cases and controls was quantified by fitting a linear model on a gene-by-gene basis [28],[29]. Of 21,911 genes examined, 693 genes exhibited case/control differences at P13, as opposed to 92 at P7, and 83 at P1 (Figure S1, Tables S2, S3, S4, see Text S1 for details of the linear model). This is in accordance with the parallel analysis described above.

We next examined changes between time points for each genotype. Overall, the number of genes differentially expressed between time points in control mice was higher by a factor of ten compared to the number of genes differentially expressed between genotypes at individual time points (Figure 3B), suggesting that the immediate post-natal period is associated with major changes in spinal cord gene expression in normally maturing mice.

### Exon-level analysis

**Figure 4:**
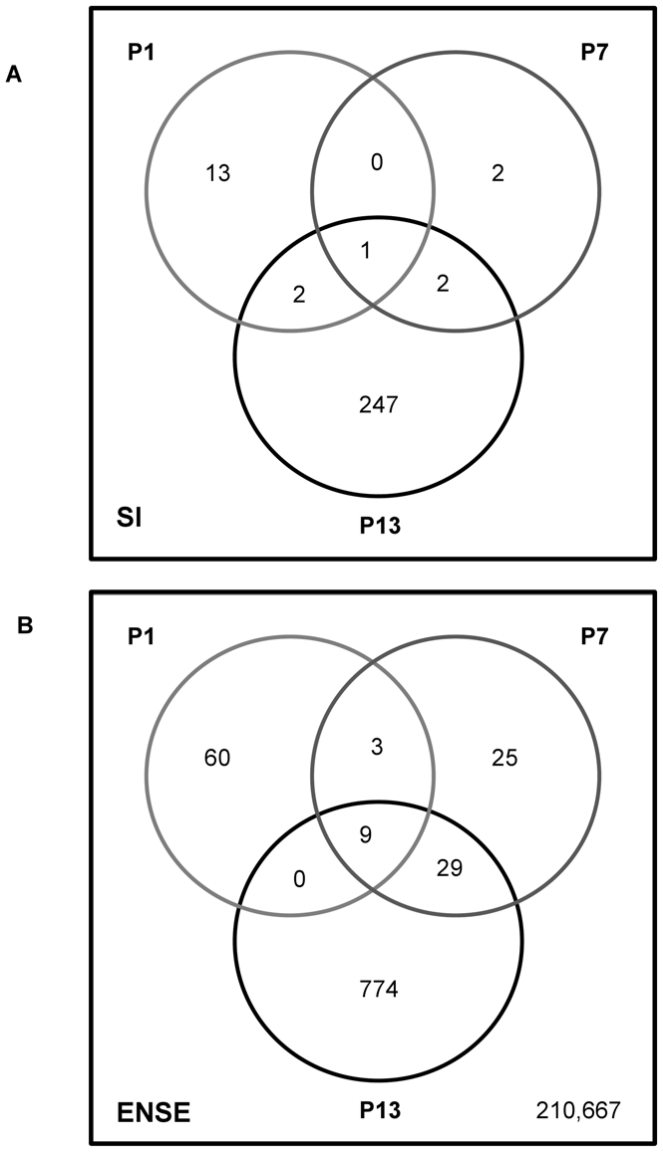
Exon-level changes are a late occurrence in SMN deficient mice. (A) Venn diagram depicting potential splicing events as evidenced by a Splicing Index |SI|>0.5. 252 alternative splicing events are present at late-symptomatic SMA mice compared to controls, but only 5 at P7 and 16 at P1. (B) Venn diagram depicting exon-level changes of Ensembl exons between genotypes at each time point investigated. At P1, P7, and P13, each of 211,567 exons was tested for differential expression between SMA and control. 812 exons were associated with disease status at P13, compared to 72 exons at P1 and 66 exons at P7.

A key aim of this study was to assess the amount of splicing variation in the spinal cord of an SMA mouse model compared to control mouse spinal cord at several time points during disease progression. Because exon-specific microarrays are relatively novel, and analysis methods have not been fully developed and validated, we used two complementary statistical approaches to investigate the number of differentially expressed exons. First we used an ANOVA test in GeneSpring (Agilent) to select exons which show a significant difference between exon-level and transcript-level signal and then calculated the splicing index (SI) [30], for these exons. The SI is the logarithm of the ratio of array probe set intensities (corresponding to expression levels of individual exons) to overall gene-level expression. So an SI value of 0 indicates equal expression of a particular exon in relation to the gene as a whole between genotypes i.e. no differential alternative splicing. Using a splicing index of |SI|≥0.5 as an arbitrary threshold we identified 252 potential alternative splicing events at the late-symptomatic stage (P13), but only 5 at the early symptomatic (P7) and 16 at the pre-symptomatic (P1) stage (Figure 4A). This initial analysis suggests that alternative splicing events are a consequence of disease progression in SMA, rather than the primary cause. Since the splicing index method is known to lead to inaccuracies if complex splicing patterns are present, such as splicing of multiple exons in one gene, and might thus underestimate the level of differential exon use present, we next performed an analysis comparing expression levels of individual exons between genotypes at each time point. The rationale for this is that each instance of differential splicing between genotypes will lead to at least one exon being differentially expressed. So the number of differentially expressed exons provides an upper bound for the number of differential splicing events. In this analysis, which we refer to as the ENSE analysis, probes were grouped into sets, each corresponding to an Ensembl exon [27]. Time point-specific differential expression between cases and controls was quantified by fitting a linear model on an exon-by-exon basis [28],[29]. The p-value cut-off for significant differences between genotypes at the exon-level was chosen to balance sensitivity with a reasonable false discovery rate (FDR), as estimated by a permutation-based analysis (Text S1 and Table S5). At a p-value threshold of 1e-4, there were 812 significantly differentially expressed exons at P13, compared to 66 at P7, and 72 at P1 (Figure 4B, Tables S6, S7, S8); a total of 211,567 exons were examined. See Materials and Methods and Text S1 for further details of this analysis.

This provided additional evidence that the vast majority of alternative splicing events occur at late-stage disease in the SMNΔ7 mouse model of SMA.

We further observed that for genes with at least one differentially expressed probe set under the ENSE annotation, but no significant gene-level change under the ENSG annotation, i.e. genes for which there is some evidence of differential splicing events, it is mostly one, and never more than two, exons that are significantly altered.

To assess whether the differentially expressed exons were associated with a particular intron type, we cross-referenced our data with a publicly available database of U12 introns, i.e. sequences recognised by the U12 snRNA containing minor spliceosome, but not the U2 snRNA containing major spliceosome [31]. Overall, the frequency of U12 introns in genes containing exons differentially expressed between genotypes was 0.19%, as opposed to the expected frequency of approximately 0.35% (0.13% at P1, 0% at P7, 0.22% at P13). Moreover, only one gene (Vash1, ENSG000000712460, Vasohibin-1) contained a differentially expressed exon directly adjacent to an U12 type intron, while all other exons were remote from U12 type introns. This analysis suggests that genes spliced by the minor spliceosome are not preferentially affected by SMA, even though components of the minor spliceosome were shown to be disproportionately affected by SMN deficiency [14].

### Expression analysis of *Smn−/−;SMN2* spinal cord

While the results obtained from the SMNΔ7mouse model afforded important insights into the dynamics of gene- and exon-level expression changes over time, to ensure that our findings were not restricted to this particular transgenic model of SMA, but applicable to SMA mouse models in general, we performed a similar analysis on the more severe but genetically less complicated *Smn−/−;SMN2* mouse model of SMA [32]. The *Smn−/−;SMN2* animals, in which complete absence of mouse Smn is rescued by the introduction of the human *SMN2* transgene, have a maximum lifespan of 6 days. Previous studies showed that at P1, there are normal motor neuron numbers, whereas at P5, there is about a 35% loss compared to litter mates with normal mouse *Smn* (*Smn+/+;SMN2*) [32]. Early synaptic abnormalities are seen from P2 in this model [33], although neuromuscular junctions appear normal at P1, indicating normal pre-symptomatic development [34]. Exon-array analysis of spinal cord harvested from pre-symptomatic (P1) and late-symptomatic (P5) animals mirrored the principal findings in the SMNΔ7 mouse at both gene and exon level. When examining gene expression in Genespring using core probe sets, more changes were present at late-symptomatic stage than at the pre-symptomatic stage (3 genes up- or down-regulated at P1, 160 genes up- or down-regulated at P5 with p≤0.05 and fold change >1.5). Even more striking was the result of the splicing index analysis, which showed 27 potential alternative splicing events at P5, but none at P1 when choosing a splicing index cut-off of 0.5.

These results showed that our findings in the SMNΔ7 mouse model were not caused by a specific effect of the SMNΔ7 transgene but are likely to be a generalised phenomenon in SMA.

### Validation of array findings by RT–PCR, western blotting, and immunohistochemistry

To validate the findings of our microarray experiments, we performed semi-quantitative and/or quantitative RT-PCR focussing on genes that showed at least one exon with differential expression at all time points (Cdkn1, Snrp1a, Chodl, Mccc2, Uspl1, ChAT, Figure S2). In addition, we performed qRT-PCR on spinal cord samples obtained from E16 embryos for some of the targets to examine whether changes were already present pre-natally. All qRT-PCR results matched the changes at gene-level seen in the array experiments, underlining the robustness of the array findings.

**Figure 5:**
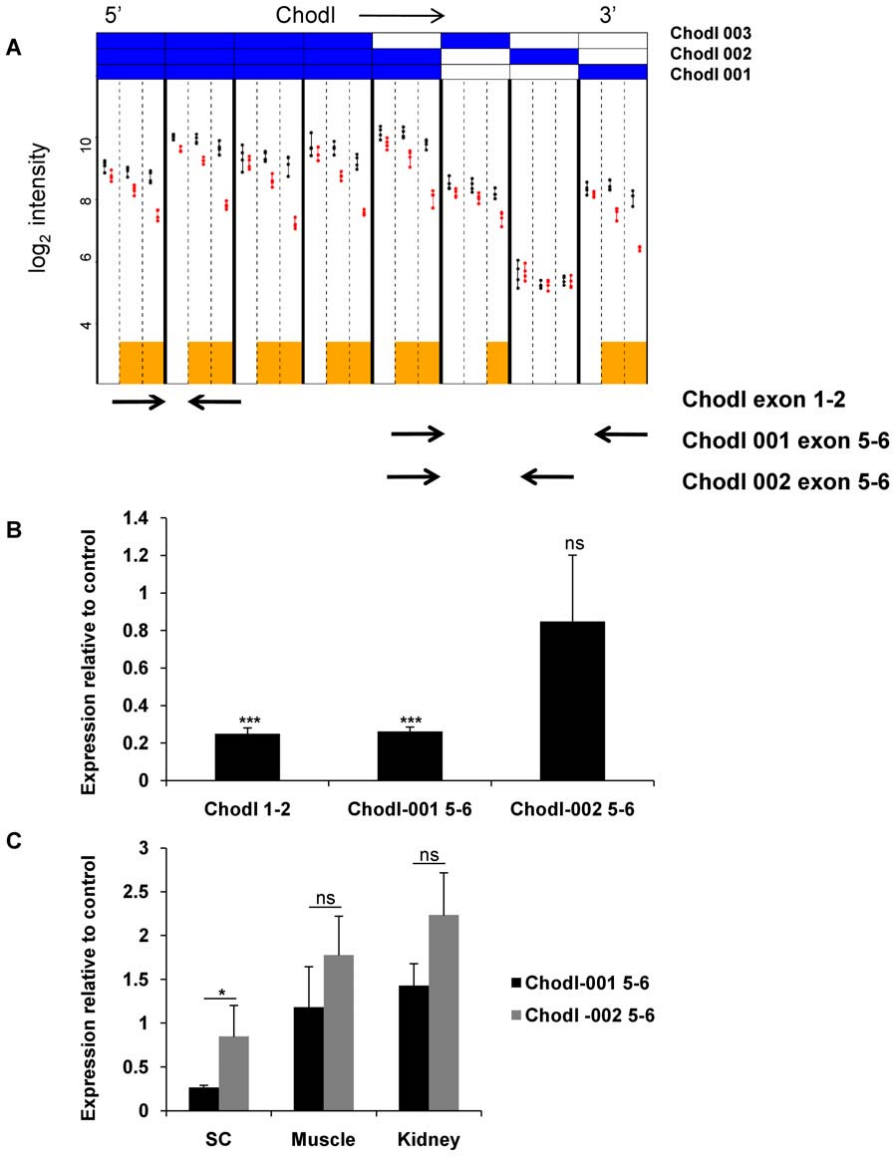
Alternative splicing of Chodl. (A) Graphical output of the exon-level analysis for Chodl. Each column, delineated by bold black lines, corresponds to the preprocessed data from a single Ensembl exon. The vertical axis displays log_2_ expression for control (black) and SMA (red) animals, with each point corresponding to an individual animal. Each column is subdivided by vertical dashed lines into time points P1, P7, and P13 (left to right). Orange boxes mark those (exon, time point) combinations that exhibit significant differential expression between cases and controls. Expression of Chodl constitutive exons is reduced progressively from P1 to P13, but there is no difference between SMA and control for the alternative terminal exon ENSMUSE00000556896 indicating an isoform shift towards Chodl-002 (ENSMUST69148) in the SMA mice. Arrows indicate location of qRT–PCR primers for validation. (B) qRT–PCR results at P13 showing marked reduction in Chodl when measured using primers located in the constitutive exons 1–2 and the terminal exon of Chodl-001, while no significant difference of alternative exon ENSMUSE00000556896 exists between control and SMA (*** = p≤1e-3). (C) The differential terminal exon usage is also evident in muscle and kidney in SMA mice, although overall transcript levels are not reduced.

Chodl, the gene encoding chondrolectin is a C-type lectin with unknown *in vivo* function. Interestingly, *in situ* hybridisation shows that this gene is highly expressed in anterior horn cells (Allen Brain Atlas http://mousespinal.brain-map.org) and might have important, if currently unknown, motor neuronal functions. We observed a progressive reduction in Chodl expression over time. Of note, the exon array data were indicative of differential expression of the 3′ end of Chodl, and validation by qRT-PCR using primers spanning both constitutive exons as well as two alternative 3′-terminal exons was in keeping with a preferential loss of the Chodl -001 isoform (ENSMUST23568) and relative sparing of Chodl-002 (ENSMUST69148) (Figure 5A and 5B). The relative reduction of Chodl expression appeared to be spinal cord specific and could not be demonstrated in either skeletal muscle or kidney. However, even these tissues showed a trend towards increased expression of the Chodl-002 isoform in SMA compared to control mice (Figure 5C). Immunohistochemistry for Chodl showed strong immunoreactivity of anterior horn cells, in keeping with the published *in situ* data. There was reduced Chodl immunoreactivity in SMA mice, but remaining anterior horn cells retained substantial Chodl staining, which indicated that the reduced Chodl expression is at least partially due to loss of motor neurons (Figure S4).

**Figure 6:**
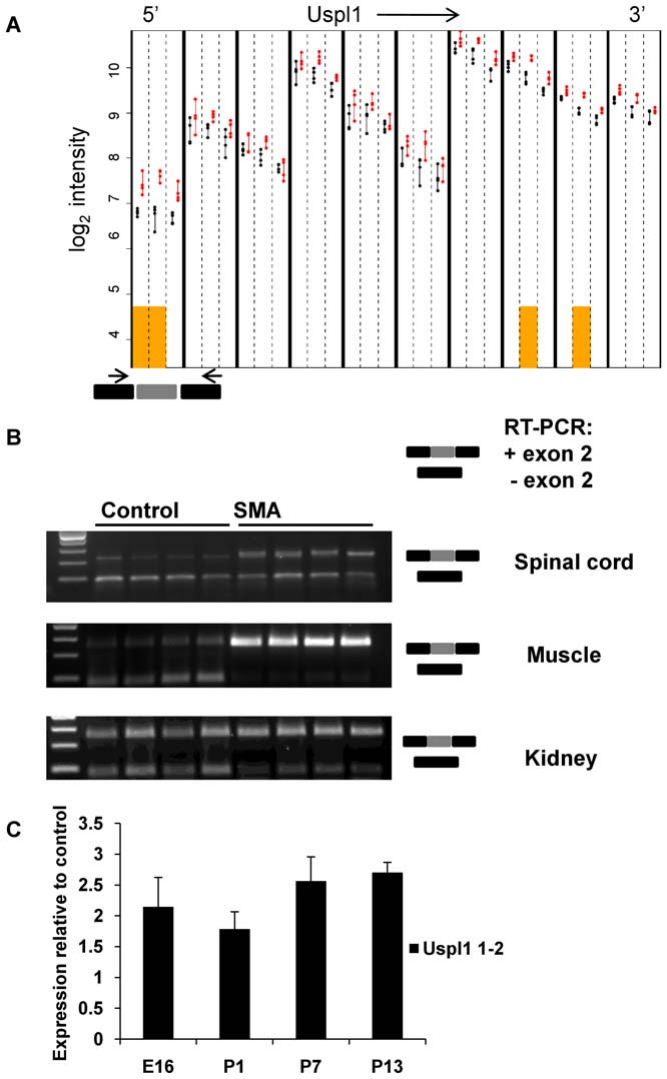
Alternative splicing of Uspl1. (A) Graphical output of exon-level analysis for Uspl1. Each column, delineated by bold black lines, corresponds to the preprocessed data from a single Ensembl exon. The vertical axis displays log_2_ expression for control (black) and SMA (red) animals, with each point corresponding to an individual animal. Each column is subdivided by vertical dashed lines into time points P1, P7, and P13 (left to right). Orange boxes mark those (exon, time point) combinations that exhibit significant differential expression between cases and controls. Expression of Uspl1 is higher in SMA mice for all exons, but this difference is more pronounced for the first exon detected by the array, which corresponds to Uspl1 exon 2 (ENSMUSE00000351955), reflecting a potential alternative splicing event. (B) Validation of the alternative splicing event by RT–PCR. Cartoon depicting primer position in Uspl1 exon 1 and 3. RT–PCR was performed on four biological replicates at P13 showing exon 2 skipping in control mice, and increased exon 2 usage in SMA mice. The difference of the exon 2+/exon 2- ratio between genotypes is tissue specific and most pronounced in muscle and less obvious in kidney. (C) qRT–PCR results for exon 2 showing increased in Uspl1 exon 2 expression in the SMA mice compared to control. The differential expression is more pronounced at symptomatic compared to pre-symptomatic stages.

Uspl1 (ubiquitin specific peptidase like 1), a gene encoding part of the ubiquitin-dependent protein degradation pathway was found to be up-regulated in SMA at all time points. In addition, Uspl1 showed a consistent change in both splicing index and Ensembl exon-level analysis of differential splicing. Uspl1 exon 2 (ENSMUSE00000351955) is a cassette exon absent in transcripts Uspl1-006 (ENSMUST00000121416) and -007 (ENSMUST00000117878). The increased use of Uspl1 exon 2 in the SMA mice led to an isoform shift with relatively higher levels of exon 2 containing transcripts Uspl1 -001-005 (Figure 6). Of note, this pattern was much more pronounced in muscle than in spinal cord, and less evident in kidney (Figure 6B). In spinal cord samples, the degree of isoform shift was more pronounced at symptomatic than at pre-symptomatic stages (Figure 6C). Immunohistochemistry for Uspl1 showed ubiquitous cytoplasmic staining in all spinal cord cells with grey matter preference. In keeping with the only mild overall increase in Uspl1 expression at mRNA level (Figure S2F), no difference in immunoreactivity was evident between genotypes Figure S4) and Western blotting showed no significant difference in the main protein isoform identified between genotypes.

Further interesting changes at all time points were detected including the whole-gene up-regulation of Snrpa1 (average 1.8 fold). Snrpa1 encodes one of the many protein components of the spliceosomal A complex [34], which is associated with the U2 snRNA. Western blotting showed a consistent small expression increase (Figure S4). While no other spliceosomal components were differentially expressed, the Snrpa1 change might reflect a compensatory response of the cell to Smn deficiency.

Another change included down-regulation of isoforms ENSMUST00000091326 and ENSMUST00000022148 of Mccc2, the gene encoding the methylcrotonoyl-CoA carboxylase beta chain, a mitochondrial enzyme involved in amino acid metabolism (Figure S2D). Interestingly, the array data suggest that, while two Mccc2 isoforms were expressed at lower levels in SMA compared to controls, a third isoform (ENSMUST00000109383) was in fact up-regulated. This was confirmed by qRT-PCR and semi-quantitative PCR (Figure S3).

### Pathway analysis

In the SMNΔ7 mouse model, the early postnatal days appeared particularly relevant to disease development. Our earlier finding of massive gene expression changes between time points in post-natal wild-type mice (Figure 3B) indicates that events relevant to disease in the SMN Δ7 mouse model coincide with transcriptome changes associated with normal post-natal development or maturation. To analyse which pathways were physiologically activated during this time, we first compared gene expression in control mice (*Smn+/+;SMN2;SMN*Δ*7*) between P1 and P7, and subsequently between P7 and P13. Using GO-Elite software, we identified pathways enriched with genes involved in spinal cord cell proliferation, axon development, oligodendrocyte development and myelination as significantly altered, reflecting physiological events during the rapid growth of the spinal cord. When the same analysis was performed for the SMA mice, a strikingly different pattern emerged. With the exception of two GO IDs pertaining to nervous system development, all physiologically activated pathways were absent in both the P1 v P7 and P7 v P13 analyses Table 3). To investigate whether this dramatic change in gene expression pathways was mirrored by a difference in proliferating spinal cord cells, we performed Western blotting and immunohistochemistry for the proliferation marker PCNA. Our preliminary results indicate that there is indeed a reduction in the number of proliferating cells in the SMA spinal cord (Figure S5). At P13, genes relating to cellular responses to DNA damage became prominent in the SMA mice, which could not be explained by a significant amount of spinal cord gliosis (Figure S5). Of note, the gene with the highest-fold change between genotypes at P13 was Cdkn1a, a cyclin dependent kinase inhibitor activated by p53 in response to DNA damage. This analysis suggested that in the SMNΔ7 mouse model there is an inhibition or a failure of activation of the normal physiological pathways of post-natal spinal cord maturation.

**Table 3:** Comparison of between time point changes in control and SMA mice using GO Elite for >50% and 3 genes changed.

**Control**		**SMA**	
**P1 versus P7**		**P1 versus P7**	
**GOID**	**GO Name**	**GOID**	**GO Name**
10456	cell proliferation in dorsal spinal cord	33269	internode region of axon
5243	gap junction channel activity	43209	myelin sheath
5452	inorganic anion exchanger activity		
33269	internode region of axon		
43209	myelin sheath		
14003	oligodendrocyte development		
5248	voltage\-gated sodium channel activity		
22829	wide pore channel activity		
**P7 versus P13**		**P7 versus P13**	
**GOID**	**GO Name**	**GOID**	**GO Name**
10456	cell proliferation in dorsal spinal cord	18198	peptidyl\-cysteine modification
6601	creatine biosynthetic process	17154	semaphorin receptor activity
6600	creatine metabolic process	48407	platelet\-derived growth factor binding
32291	ensheathment of axons in the CNS	5248	voltage\-gated sodium channel activity
5243	gap junction channel activity	9065	glutamine family amino acid catabolic process
5452	inorganic anion exchanger activity		
33269	internode region of axon		
43209	myelin sheath		
22010	myelination in the central nervous system		
14003	oligodendrocyte development		
33270	paranode region of axon		
19911	structural constituent of myelin sheath		
5248	voltage\-gated sodium channel activity		
22829	wide pore channel activity		
		**P7 versus P13 20%-49% changed**	
		**GOID**	**GO Name**
		77	DNA damage checkpoint
		32508	DNA duplex unwinding
		32392	DNA geometric change
		6270	DNA replication initiation
		6268	DNA unwinding during replication

Only a selection of GOIDs changed 20%–49% in SMA mice is shown.

## Discussion

In this study we undertook a detailed assessment of transcriptional changes over time in the spinal cord of a commonly used severe mouse model of SMA, using time points correlated with key phenotypic and pathological changes. We identified alterations in a subset of genes involved in post-natal neurodevelopmental pathways in SMA, and showed that splicing alterations are only a late occurrence in disease.

Survival, weight development and motor phenotype of our SMNΔ7 mouse colonies were similar to that described by others [26],[35], with a change in outward appearance and development of motor deficits apparent at P7. Recent studies have identified morphological changes at the neuromuscular junction as early events in SMA [33],[35] with neurofilament accumulation occurring as early as P5 in the SMNΔ7 model. This structural change at the distal end of the motor neuron is closely followed in our study by a significant loss of large motor neurons at P7, indicating that although synaptic changes are the earliest identified feature of SMA it ultimately is a disease of the entire lower motor neuron. Of note, motor neuron loss was present to a similar degree across the entire spinal cord, in contrast to the previous finding of a rostral-caudal gradient with relative sparing of the lumbar region [35]. Motor neuron loss at P7 was reflected in the reduction at transcript level of choline acetyl transferase (ChAT), the key enzyme in motor neuronal synthesis of acetylcholine (Figure S2E). This finding, as well as that of reduced levels of Chodl mRNA, which seems to be highly expressed in anterior horn cells, shows that even though whole spinal cord was used for the array, important cell-type specific changes were detectable.

The key finding of this study is that alternative splicing events are a late occurrence in SMA, and are therefore unlikely to contribute to early disease pathogenesis. Zhang *et al.* [15] described widespread splicing abnormalities in several tissues including the spinal cord at end-stage disease in the same mouse model employed in this study and attributed this finding to dysfunction of the spliceosome secondary to SMN deficiency. Importantly, even though the time point of analysis is not absolutely identical to ours, there is considerable overlap between Zhang *et al.*'s data set obtained at P11 and our P13 data set, when raw data are analysed in the same way (Figures S6, S7; Tables S1, S9, S10, S11). However, it remains unclear from these data whether splicing abnormalities had preceded the onset of symptoms, as would be predicted from the crucial role of SMN in spliceosome assembly, particularly in embryonic and early post-natal development [13]. In addition, the presence of widespread splicing defects in organs other than the spinal cord is difficult to interpret in the light of apparent tissue specificity of the disease if splicing abnormalities are indeed thought to be relevant to the mechanism of motor neuron degeneration. To determine the degree of variation in splicing between SMA and control mice, we utilised the splicing index, but also examined changes at individual exons as a measure of the maximum number of alternative splicing events present. The absolute number of changes found in late-symptomatic mice was not large given the large number of exons examined (>200,000). When the same analysis was performed, comparing between genotypes at pre-symptomatic and early symptomatic time points, only very few exon-level changes were present. Importantly, we were able to confirm a similar pattern of exon expression changes in the more severe Smn−/−;SMN2 mouse model, corroborating our main finding in the SMNΔ7 mice. Overall, our data indicate that the majority of splicing changes are not a direct consequence of SMN deficiency, but rather a consequence of disease progression, probably representing physiological isoform-shifts in response to cell stress. There is evidence that oxidative stress can induce shifts in alternative splicing, and that neurons may be more vulnerable to this process than other cells [36]. We would therefore argue that SMN deficiency to the degree observed in either the SMNΔ7 or Smn−/−;SMN2 mouse, although associated with severe reduction in snRNP assembly capacity *in vitro* [14], does not lead to a systemic breakdown of splicing fidelity *in vivo* until the disease is well established.

While our results do not support the hypothesis that widespread, systematic splicing abnormalities cause SMA, we cannot rule out the possibility that splicing of one or several transcripts is critically affected by SMN deficiency, and that the few splicing changes observed early in our mice contribute to SMA pathogenesis, followed by a cascade of loss of splicing fidelity or secondary effects. In fact, at least one of the genes identified by our array (Uspl1) is differentially spliced between SMA and control mice even at the embryonic phase, albeit to a lesser degree than at the symptomatic stages. Of note, the isoform shift observed in this particular gene is more pronounced in muscle than in spinal cord, and less marked in kidney, an organ not affected by SMA pathology.

Independent of whether or not splicing changes are ultimately responsible for SMA disease initiation, our study identified several pathways that might shed light on SMA pathogenesis and disease progression. Analysis of transcriptional changes between genotypes in this study took place on a background of large scale physiological changes between time points, reflecting rapid neuronal development in the early post-natal days. Analysis of the changes between time points identified several pathways related to normal neuronal development. Surprisingly, in the SMA mice the majority of physiological transcriptional changes seen in control mice were absent even between the early time points P1 and P7. This finding not only indicates that abnormal post-natal neuronal development might underlie early events in SMA but might explain general delayed growth and failure to thrive in the SMA mice.

In this study, we examined gene expression in the entire spinal cord and are unable to distinguish which cell types contribute most to expression changes, even though changes that plausibly originate from motor neurons, such as ChAT and Chodl, are clearly present. The neurodevelopmental pathways identified as altered in our study can be associated with other cell types, including oligodendrocytes and cells in the posterior spinal cord. While this is a preliminary finding, it clearly warrants further studies examining the role of non-motor neuronal cells in SMA. Human autopsy cases indicate involvement of sensory neurons in the dorsal root ganglia, Clarke's column and the thalamus in SMA [37]–[40], although the majority of studies were undertaken before the molecular diagnosis of SMA was available. More recent clinical studies showed that severe SMN-related SMA is associated with widespread neuronal degeneration, including sensory pathways [41]. Subtle sensory neuron abnormalities have also been detected in a severe mouse model of SMA [42]. To our knowledge, however, there is no study systematically investigating the role of non-motoneuronal cells in SMA spinal cord.

In conclusion, our data show that alternative splicing events predominantly occur late in SMA, while alterations of post-natal neurodevelopmental pathways precede overt symptom onset. Further studies should continue to focus on the role of SMN in the post-natal maturation and development of the neuromuscular system including spinal cord motor neurons.

## Materials and Methods

### Mice

Transgenic *Smn^+/−^;SMN2;SMN*Δ*7* [26] mice were maintained as heterozygous breeding pairs in standard animal facilities in Oxford. Homozygous *Smn^−/−^; SMN2; SMN*Δ*7* mice reached the disease end-point by post-natal day 13 (P13). Five mice of each genotype were sacrificed at age P1, P3, P5, P7, P9, P11 and P13 for motor neuron counts, and 4 mice of each genotype at P1, P7 and P13 for RNA and protein extraction.

Mice were genotyped using DNA extracted from tail-tips and standard PCR procedures.

For motor neuron counts, mice were terminally anaesthetised with i.p. pentobarbitone and transcardially perfused with phosphate-buffered saline (PBS) followed by 4% paraformaldehyde (PFA) in PBS.

For RNA and protein extraction, mice were killed by i.p. injection of pentobarbitone.

*Smn^+/−^;SMN2* mice (Jackson labs strain no. 005024) were maintained as heterozygote breeding pairs under standard SPF conditions in animal care facilities in Edinburgh [43]. Litters produced from SMA colonies were retrospectively genotyped using standard PCR protocols (JAX® Mice Resources).

All animal breeding and procedures were performed in accordance with Home Office and University guidelines.

### Motor neuron counts and immunohistochemistry

Spinal cords were dissected, post-fixed in 4% PFA for 2 hours, cryoprotected in 30% sucrose overnight, embedded in OCT medium and rapidly frozen in liquid nitrogen-cooled isopentane. 20 µm thick horizontal sections were cut on a cryostat and stained with 0.5% Cresyl violet with 0.04% acetic acid. A minimum of 30 non-adjacent sections covering the entire spinal cord segment of interest were scrutinised for large, polygonal, Nissl positive cells in the ventral horn of the spinal cord anterior to the central canal. Only cells with a clearly present nucleolus were counted to avoid double counting of neurons. Motor neuron counts were performed blinded to genotype. At P13, cords were macroscopically divided into cervical, thoracic and lumbar segments using the cervical and lumbar enlargements as landmarks. For the time-course, only lumbar spinal cord was utilised.

6 µm thick paraffin section were cut and stained with standard Haematoxylin and Eosin. For immunohistochemistry, sections were incubated with the primary antibody (mouse anti-SMN antibody (1∶320, BD Transduction lab), rabbit anti-Uspl1 (1∶600, Santa Cruz), mouse anti-Chodl (1∶200, abcam), rabbit anti-PCNA (1∶2500, abcam), goat anti-Chat (1∶400, Chemicon) for 40 minutes at room temperature or at 4°C overnight. Antibody binding was visualised using a Dako REAL EnVision kit according to manufacturer's instructions.

Immunohistochemistry was carried out on several sections taken from two different paraffin blocks for two animals per genotype. Representative images are shown.

### RNA isolation and microarray

Whole spinal cords were rapidly dissected and snap-frozen on dry ice. RNA was extracted using the Qiagen RNeasy Mini RNA extraction kit according to the manufacturer's instructions.

The quality and RNA integrity was assessed on a BioAnalyzer; all samples had a RNA Integrity Number (RIN)≥9 (Agilent Laboratories, US). 1 ug starting RNA was ribosome depleted using the Ribominus Human/MouseTranscriptome Isolation kit (Invitrogen). Labelled sense ssDNA for hybridization was generated with the Affymetrix GeneChip WT sense target labelling and control reagents kit (Affymetrix, UK) according to the manufacturer's instructions. Sense ssDNA was fragmented and the distribution of fragment lengths was measured on the BioAnalyzer. The fragmented ssDNA was labelled and hybridized to the Affymetrix GeneChip Mouse Exon 1.0 ST Array (Affymetrix). Chips were processed on an Affymetrix GeneChip Fluidics Station 450 and Scanner 3000.

### Microarray gene expression and pathway analysis

For the gene-level analysis, core probe sets which map to the same transcript cluster were grouped together and RMA (Robust multi-array analysis) [44] normalised in GeneSpring GX10.1.02. Differentially expressed genes were identified using an unpaired t-test; selecting genes with 1) a p-value less than or equal to an arbitrary threshold of 0.05 and 2) a fold change difference between genotypes ≥1.5. The selected genes were sorted according to gene ontology using GenMAPP's GO-Elite (http://www.genmapp.org/go_elite/go_elite.html). Only MAPPFinder ontologies with ≥3 genes changing and a permuted p-value of ≤0.05 were reported.

### Splicing index

At the exon level, core probe sets were PLIER (Probe Logarithmic Intensity Error) normalised in GeneSpring GX 10.1.02 (Agilent). Transcript probe sets that had detection above background (DABG) p-value≤0.05 in both SMA and control groups were retained. An ANOVA test was used to identify significant differences between exon-level signal and transcript level signal. As recommended in the Affymetrix White Paper [44],[45], exon level probe sets exhibiting exon-to-transcript intensity ratios >5 were excluded from the ANOVA (where log_2_[exon-to-transcript ratio] = log_2_[exon expression]−log_2_[transcript expression]). This filter removed probes with high background and cross-hybridisation potential. The p-value threshold for the ANOVA was selected to control for a false discovery rate of 0.05 using the Benjamini-Hochberg multiple testing procedure [46]. For exons selected on the basis of the ANOVA, the spicing index, SI (log_2_[exon-to-transcript expression ratio]), was calculated and used as a measure of differential splicing between genotypes. See [45],[47] for further details. A significantly differentially spliced exon was defined to be one having both an FDR-controlled ANOVA p-value≤0.05 and |SI|≥0.5 (on the log scale, corresponding to a fold change up or down of approximately 1.4 in the absolute exon/transcript ratio between genotypes).

### Ensembl exon and Ensembl gene expression analysis

CEL files were preprocessed using RMA without background correction (see Text S1). Publicly available custom chip-definition files (CDFs) (http://brainarray.mbni.med.umich.edu/Brainarray/Database/CustomCDF/CDF_download.asp) were used to group probes into sets. Parallel analyses, based on two different CDFs were performed. The first CDF, referred to as ENSE, defines a probe set for each Ensembl exon. The second, ENSG, defines a probe set for each Ensembl gene [27].

There were 211,567 and 21,911 probe sets for the ENSE and ENSG analyses respectively. At each probe set, a linear model was fitted using the limma package (version 2.16.5), to quantify evidence of genotype differences within each time point (see Text S1 for details).

### False discovery rate analysis

A permutation-based analysis was conducted to estimate the FDR at a variety of p-value thresholds (see Text S1 and Table S5).The p-value thresholds selected were 1e-4 for ENSE, and 1e-3 for ENSG, as these thresholds controlled the FDR at a reasonably low level.

Statistical analyses were performed using R [28], version 2.8.1.

### Protein extraction

Snap frozen whole spinal cords were homogenised in RIPA lysis buffer (50 mM Tris-Cl, pH 7.5, 150 mM NaCl, 0.1% (w/v) SDS, 1% (v/v) sodium deoxycholate, 1% (v/v) TX-100) and 1% (v/v) protease inhibitors by sonication at 50% output for 15 sec. Homogenates were chilled on ice for 20 min and clarified by centrifugation at 15,800 g for 20 min at 4°C.

### Immunoblotting

Protein samples (50 µg/well) were electrophoresed through 12% SDS polyacrylamide gels and transferred to 0.2 µm nitrocellulose membranes (Millipore). Membranes were blocked with 5% (w/v) milk powder in TBS-T, pH 8.0, for 1 hr and incubated with the primary antibody (mouse anti-SMN (1∶1,000, BD Transduction labs), mouse anti-actin (1∶1,000, Abcam), rabbit anti-SNRPA1 (1∶1000, abcam), goat anti-CHAT (1∶500, Chemicon), rabbit anti-USPL1 (1∶500, Santa Cruz), rabbit anti-PCNA (1∶200, abcam) in 3% (w/v) BSA in TBST overnight at 4°C. Blots were probed with HRP-conjugated antibodies (1∶10,000, Amersham) and developed using ECL reagents (Amersham).

Western blots were repeated three times on biological replicates, and representative blots are shown.

### RT–PCR and qRT–PCR

RNA was reverse transcribed into cDNA using random hexamer primers (Invitrogen) and Expand Reverse Transcriptase (Roche) under standard conditions.

RT-PCR was performed using Taq DNA Polymerase (Sigma) or Expand High Fidelity PCR system (Roche). Real-time PCR was performed using Fast SYBR Green chemistry (Applied Biosystems) and a StepOnePlus Real-time PCR machine (Applied Biosystems). Real-time PCR Primers were designed with Primer Express software (Applied Biosystems). Primer concentrations were optimised to yield low Ct values and minimal primer dimer formation (commonly 300 nM for both forward and reverse primers). GAPDH was used as the endogenous control, as there was no differential expression between genotype in the array and in qRT-PCR experiments. All primer pairs were tested to have similar amplification efficiency to GAPDH when tested on serial cDNA dilutions over 4 log. Fold change was calculated using standard ΔΔCt calculations. The average fold change per time point was calculated from four biological replicates at each time point, and an unpaired two-tailed t-test was used to test for significantly different gene expression at each time-point. Error bars represent the standard deviation of the mean.

## Supporting Information

Figure S1 Gene level expression changes of Ensembl Genes. Venn diagram showing the number of differentially expressed genes at different time points with p≤1e-3.
(0.22 MB TIF)

Figure S2 Array validation by qRT-PCR. Quantitative RT-PCR was carried out for all time points on the gene displaying the highest fold-change in late-symptomatic mice, but no change at the pre-symptomatic stage (A: Cdkn1a) as well as several targets found to be differentially expressed at several time points in the exon array ENSE analysis (B–F: Chodl, Snrp1a, Mccc2, ChAT, Uspl1). Genes showing differential expression at P1 were also examined at embryonic stage E16. Expression is shown relative to control animals. GAPDH was used as the endogenous control. All qRT-PCR results are in agreement with the expression change predicted by the array. Error bars show the standard deviation of the mean for both 4 control and 4 SMA animals per time point. An unpaired t-test was performed between genotypes to test for significance (* = p≤0.05, ** = p≤0.01,*** = p≤1e-3).
(0.48 MB TIF)

Figure S3 Differential expression of Mccc2 isoforms. (A) Graphical output of exon array for Mccc2 (analogous to the graphical outputs of exon array data in the main manuscript). (B) At P13, qRT-PCR across exons present in isoforms Mccc2-201 (ENSMUST00000091326) and Mccc2-202 (ENSMUST00000022148) shows reduced expression in SMA compared to control (*** p≤1e-3, unpaired t-test). (C) While the reduced expression level of Mccc2-201 and Mccc2-202 is not apparent on semi-quantitative RT-PCR, the Mccc2-203 isoform ENSMUST00000109383 shows increased expression in SMA.
(0.83 MB TIF)

Figure S4 Validation of array findings at protein level. (A) Immunohistochemistry for Chodl on spinal cord sections of P13 control (A,C) and SMA (B,D) mice shows reduced Chodl immunoreactivity in the ventral horn of SMA mice, but no complete loss of Chodl from remaining anterior horn cells. Similar results are obtained for Chat in control (E,G) and SMA (F,H) mice. Both Chodl and Chat preferentially stain large anterior horn cells. Staining for Uspl1 (I,J) shows ubiquitous cytoplasmic Uspl1 expression with preference of the grey matter. (B) Chodl immunohistochemistry on adult human spinal cord shows very specific labelling of motor neurons in the ventral horn, supporting the importance of Chodl for motor neurons. (C) Western blotting of P13 spinal cord lysates shows reduced Smn and Chat protein levels, minimal increase of Snrpa1 and no overall difference in Uspl1. The Uspl1 1 antibody detected multiple bands in keeping with several known Uspl1 isoforms. MW, molecular weight in kDa. Scale bars 100 µm.
(5.48 MB TIF)

Figure S5 Markers of spinal cord proliferation and gliosis. (A) GFAP immunohistochemistry of control (A,C) and SMA (B,D) mice shows no significant difference in spinal cord gliosis at P13. (B) Western blotting for the cell proliferation marker PCNA (Proliferating Cell Nuclear Antigen antibody) shows a decrease in SMA. (C) The specificity of the antibody is shown by staining of rostral migratory stream cells [(A) no primary antibody, (B) rabbit anti-PCNA 1∶2500] in mouse brain. (D) The entral canal ependymal zone contains several PCNA positive cells in control (A,C,E), but not in SMA mice (B,D,F). GFAP, glial fibrillary acidic protein.
(6.13 MB TIF)

Figure S6 Effect of RMA background correction. RMA background correction applies a smooth, monotonic transformation from raw probe intensities to corrected probe intensities (this figure displays this transformation for a single exon array). The function is linear for medium-to-high intensities, but tends to stretch out the low-intensity range (the figure is annotated with a two-fold interval that is mapped to a ten-fold interval).
(0.25 MB TIF)

Figure S7 Comparison of P13 data set and Zhang et al data set. This figure (ENSE (A), ENSG (B)) compares the log_2_(case/control fold change) across studies. Only probe sets that are significantly differentially expressed in at least one study are included. There is clearly a degree of concordance between the two studies at these probe sets. In particular, the directionality of differential expression is extremely consistent across studies (Table S2).
(0.63 MB TIF)

Table S1 P13 gene level changes SMA vs control, fold change >1.5, P≤0.05.
(0.22 MB DOC)

Table S2 ENSG analysis P1. Differentially expressed genes at P1 under the ENSG annotation. ENSG data; “+” indicates over-expression of cases relative to controls.
(0.01 MB CSV)

Table S3 ENSG analysis P1. Differentially expressed genes at P1 under the ENSG annotation.
(0.01 MB CSV)

Table S4 ENSG analysis P7. Differentially expressed genes at P7 under the ENSG annotation.
(0.01 MB CSV)

Table S5 ENSG analysis P13. Differentially expressed genes at P13 under the ENSG annotation.
(0.06 MB CSV)

Table S6 ENSE analysis P1. Differentially expressed exons at P1.
(0.01 MB CSV)

Table S7 ENSE analysis P7. Differentially expressed exons at P7.
(0.01 MB CSV)

Table S8 ENSE analysis P13. Differentially expressed exons at P13.
(0.09 MB CSV)

Table S9 ENSG comparison P13 vs Zhang et al. Probe sets that are case/control differentially expressed (p≤1e-3) under the ENSG annotation in at least one of (a) the P13 data, (b) Zhang's spinal cord data. Along with the exon/gene IDs and the number of exons assayed in each gene, the tables include p-values, log_2_(fold change), and signed fold change.
(0.14 MB CSV)

Table S10 ENSE comparison P13 vs Zhang et al. Probe sets that are case/control differentially expressed (p≤1e-4) under the ENSE annotation in at least one of (a) the P13 data, (b) Zhang's spinal cord data. Along with the exon/gene IDs and the number of exons assayed in each gene, the tables include p-values, log_2_(fold change), and signed fold change.
(0.25 MB CSV)

Text S1 Statistical analysis of microarray findings; comparison of the P13 dataset with Zhang et al; primer sequences.
(0.09 MB PDF)
