# Supplementary figures and images for: Alternative Splicing Events Are a Late Feature of Pathology in a Mouse Model of Spinal Muscular Atrophy
Source: PLoS Genet. 2009 Dec 18;5(12):e1000773. doi: 10.1371/journal.pgen.1000773 (PMC2787017; doi:10.1371/journal.pgen.1000773)

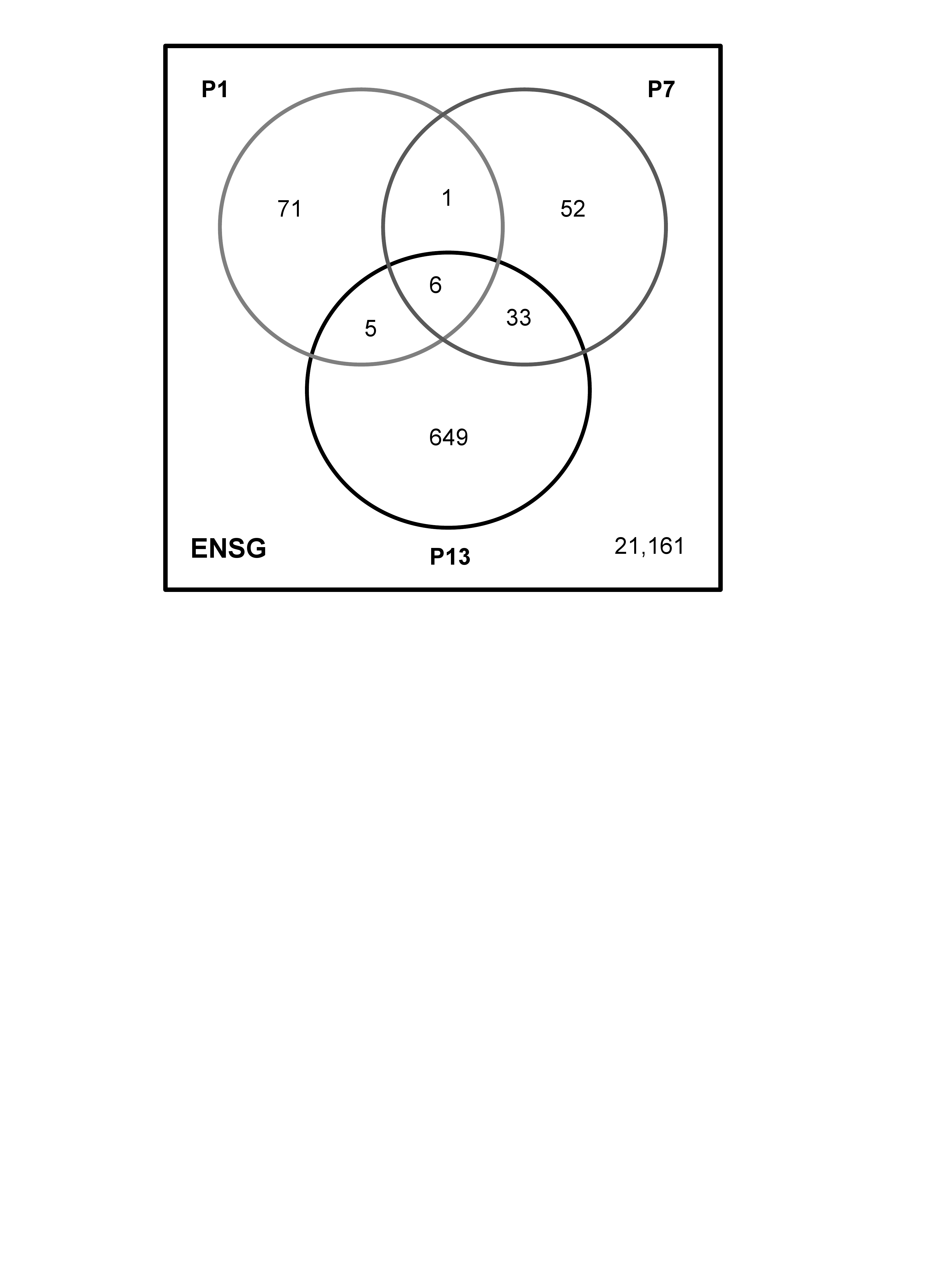

Supplement: Figure S1 — Gene level expression changes of Ensembl Genes. Venn diagram showing the number of differentially expressed genes at different time points with p≤1e-3. (0.22 MB TIF) [file pgen.1000773.s001.tif]

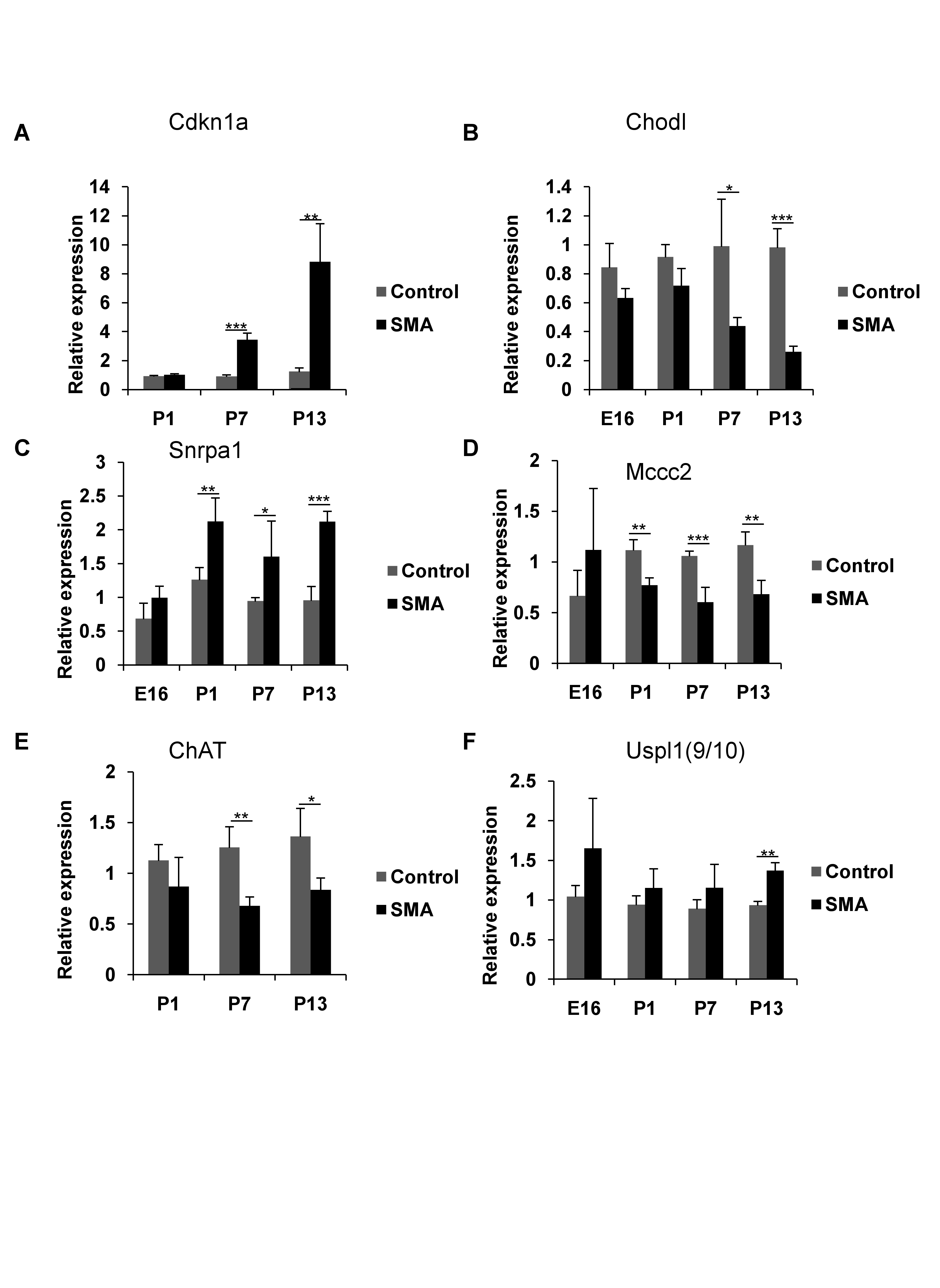

Supplement: Figure S2 — Array validation by qRT-PCR. Quantitative RT-PCR was carried out for all time points on the gene displaying the highest fold-change in late-symptomatic mice, but no change at the pre-symptomatic stage (A: Cdkn1a) as well as several targets found to be differentially expressed at several time points in the exon array ENSE analysis (B–F: Chodl, Snrp1a, Mccc2, ChAT, Uspl1). Genes showing differential expression at P1 were also examined at embryonic stage E16. Expression is shown relative to control animals. GAPDH was used as the endogenous control. All qRT-PCR results are in agreement with the expression change predicted by the array. Error bars show the standard deviation of the mean for both 4 control and 4 SMA animals per time point. An unpaired t-test was performed between genotypes to test for significance (* = p≤0.05, ** = p≤0.01,*** = p≤1e-3). (0.48 MB TIF) [file pgen.1000773.s002.tif]

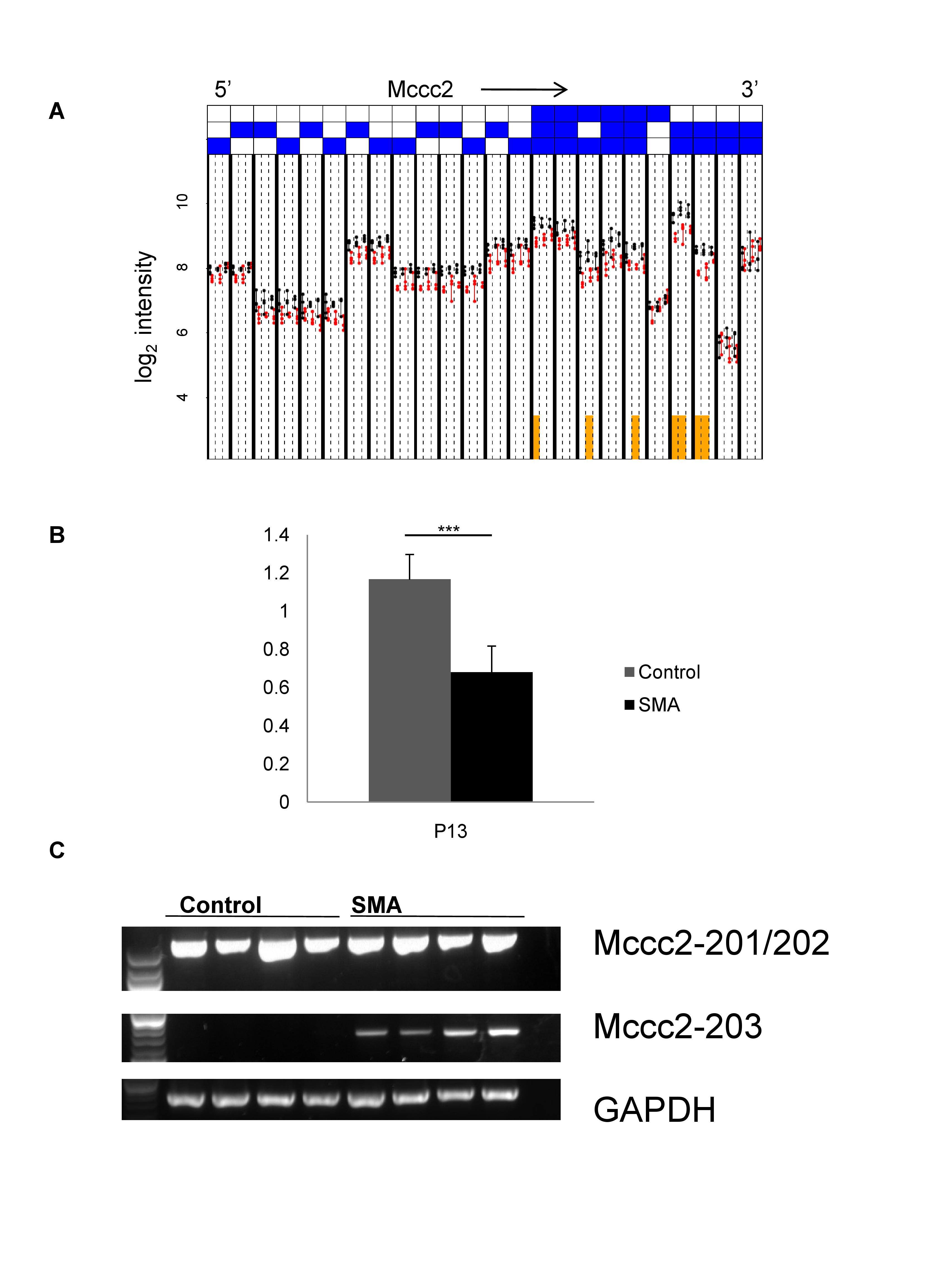

Supplement: Figure S3 — Differential expression of Mccc2 isoforms. (A) Graphical output of exon array for Mccc2 (analogous to the graphical outputs of exon array data in the main manuscript). (B) At P13, qRT-PCR across exons present in isoforms Mccc2-201 (ENSMUST00000091326) and Mccc2-202 (ENSMUST00000022148) shows reduced expression in SMA compared to control (*** p≤1e-3, unpaired t-test). (C) While the reduced expression level of Mccc2-201 and Mccc2-202 is not apparent on semi-quantitative RT-PCR, the Mccc2-203 isoform ENSMUST00000109383 shows increased expression in SMA. (0.83 MB TIF) [file pgen.1000773.s003.tif]

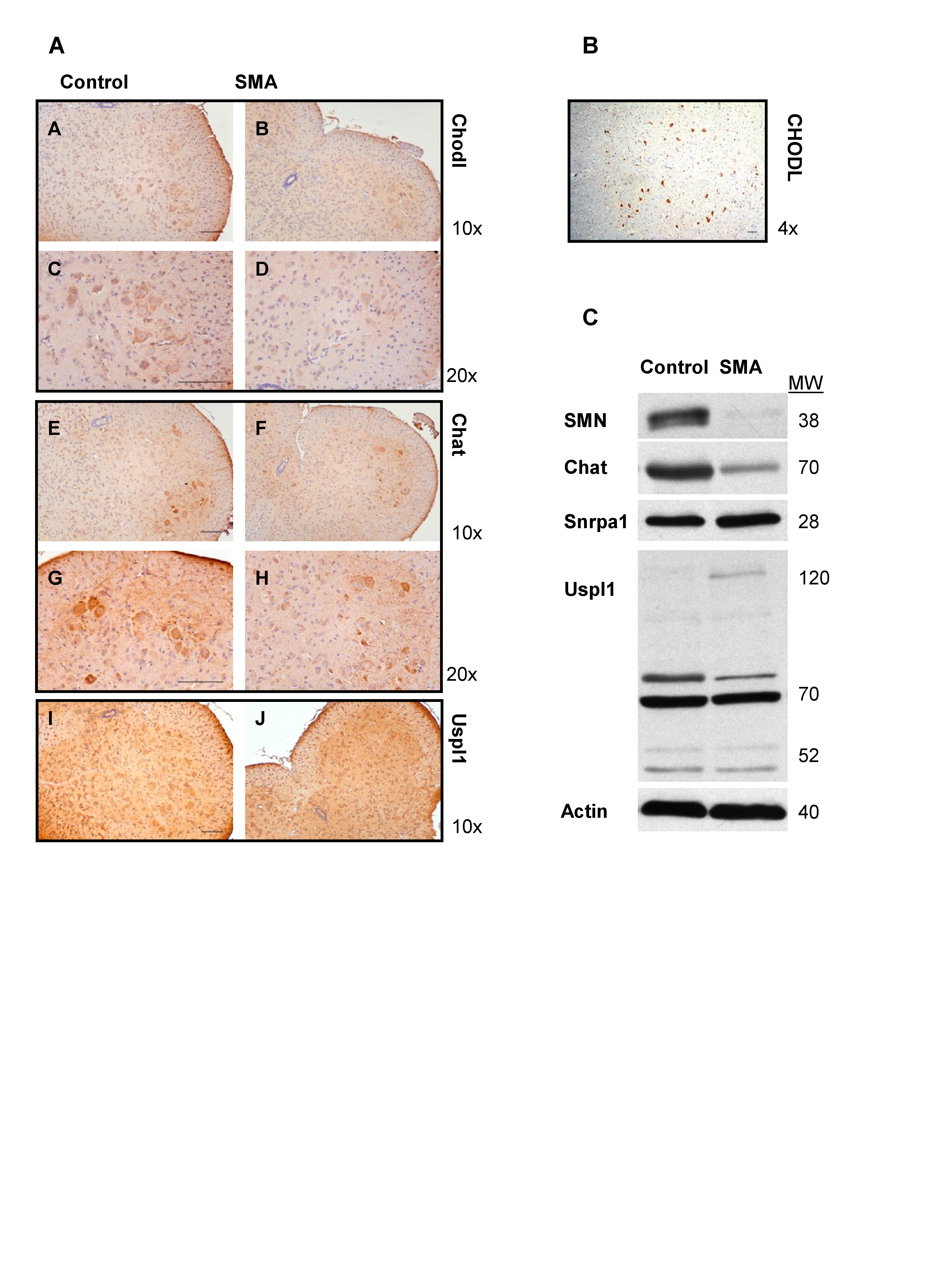

Supplement: Figure S4 — Validation of array findings at protein level. (A) Immunohistochemistry for Chodl on spinal cord sections of P13 control (A,C) and SMA (B,D) mice shows reduced Chodl immunoreactivity in the ventral horn of SMA mice, but no complete loss of Chodl from remaining anterior horn cells. Similar results are obtained for Chat in control (E,G) and SMA (F,H) mice. Both Chodl and Chat preferentially stain large anterior horn cells. Staining for Uspl1 (I,J) shows ubiquitous cytoplasmic Uspl1 expression with preference of the grey matter. (B) Chodl immunohistochemistry on adult human spinal cord shows very specific labelling of motor neurons in the ventral horn, supporting the importance of Chodl for motor neurons. (C) Western blotting of P13 spinal cord lysates shows reduced Smn and Chat protein levels, minimal increase of Snrpa1 and no overall difference in Uspl1. The Uspl1 1 antibody detected multiple bands in keeping with several known Uspl1 isoforms. MW, molecular weight in kDa. Scale bars 100 µm. (5.48 MB TIF) [file pgen.1000773.s004.tif]

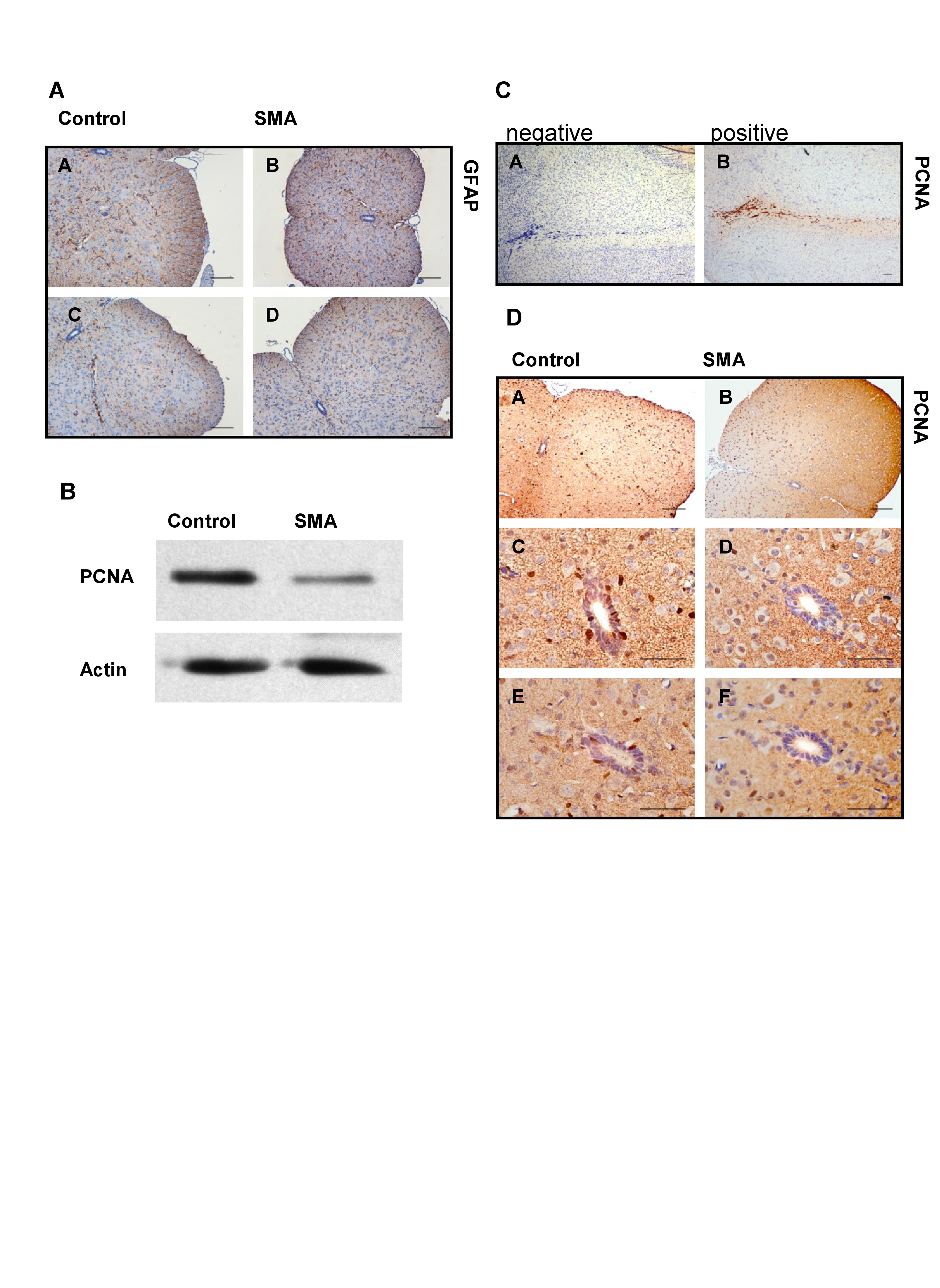

Supplement: Figure S5 — Markers of spinal cord proliferation and gliosis. (A) GFAP immunohistochemistry of control (A,C) and SMA (B,D) mice shows no significant difference in spinal cord gliosis at P13. (B) Western blotting for the cell proliferation marker PCNA (Proliferating Cell Nuclear Antigen antibody) shows a decrease in SMA. (C) The specificity of the antibody is shown by staining of rostral migratory stream cells [(A) no primary antibody, (B) rabbit anti-PCNA 1∶2500] in mouse brain. (D) The entral canal ependymal zone contains several PCNA positive cells in control (A,C,E), but not in SMA mice (B,D,F). GFAP, glial fibrillary acidic protein. (6.13 MB TIF) [file pgen.1000773.s005.tif]

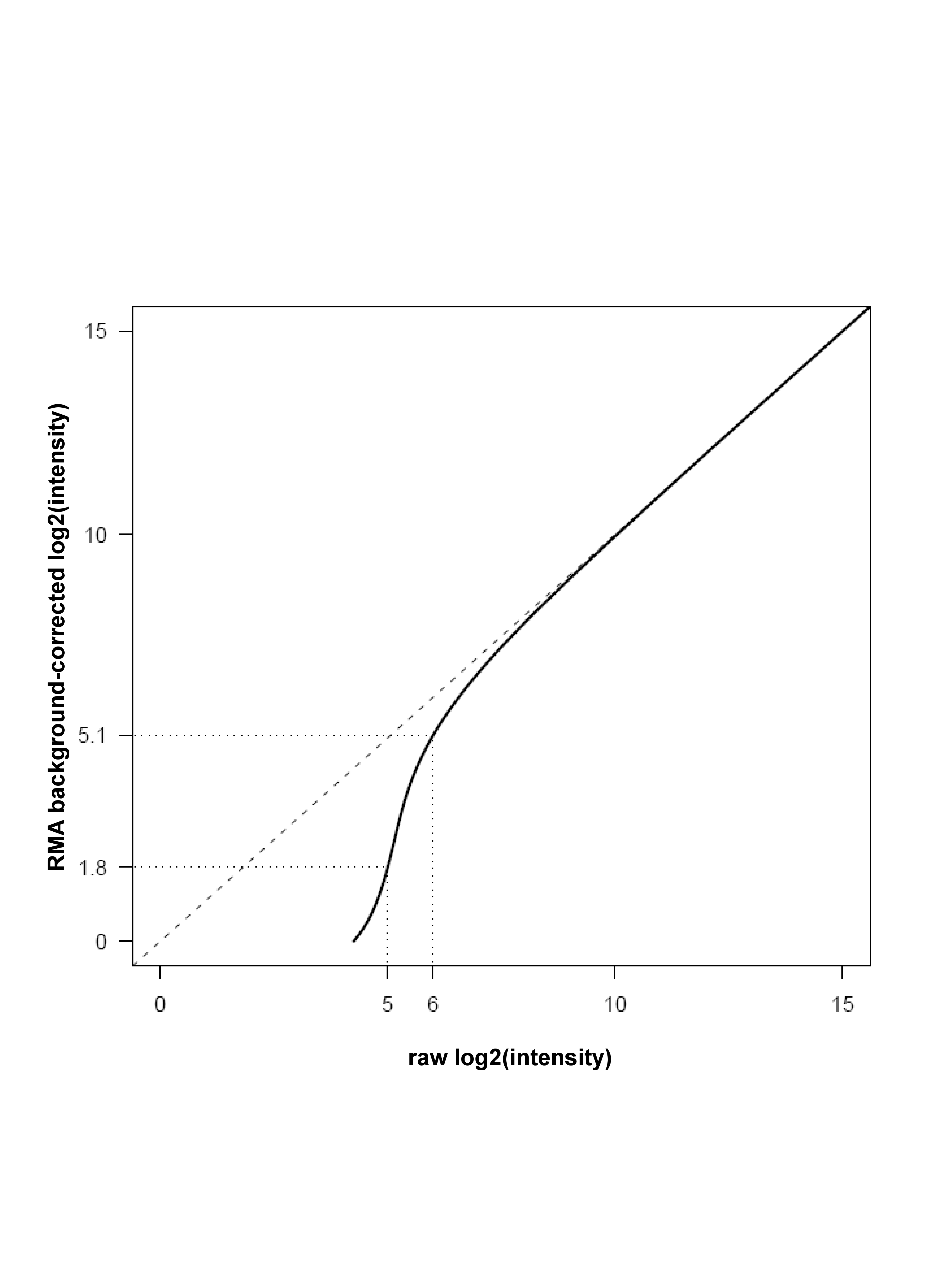

Supplement: Figure S6 — Effect of RMA background correction. RMA background correction applies a smooth, monotonic transformation from raw probe intensities to corrected probe intensities (this figure displays this transformation for a single exon array). The function is linear for medium-to-high intensities, but tends to stretch out the low-intensity range (the figure is annotated with a two-fold interval that is mapped to a ten-fold interval). (0.25 MB TIF) [file pgen.1000773.s006.tif]

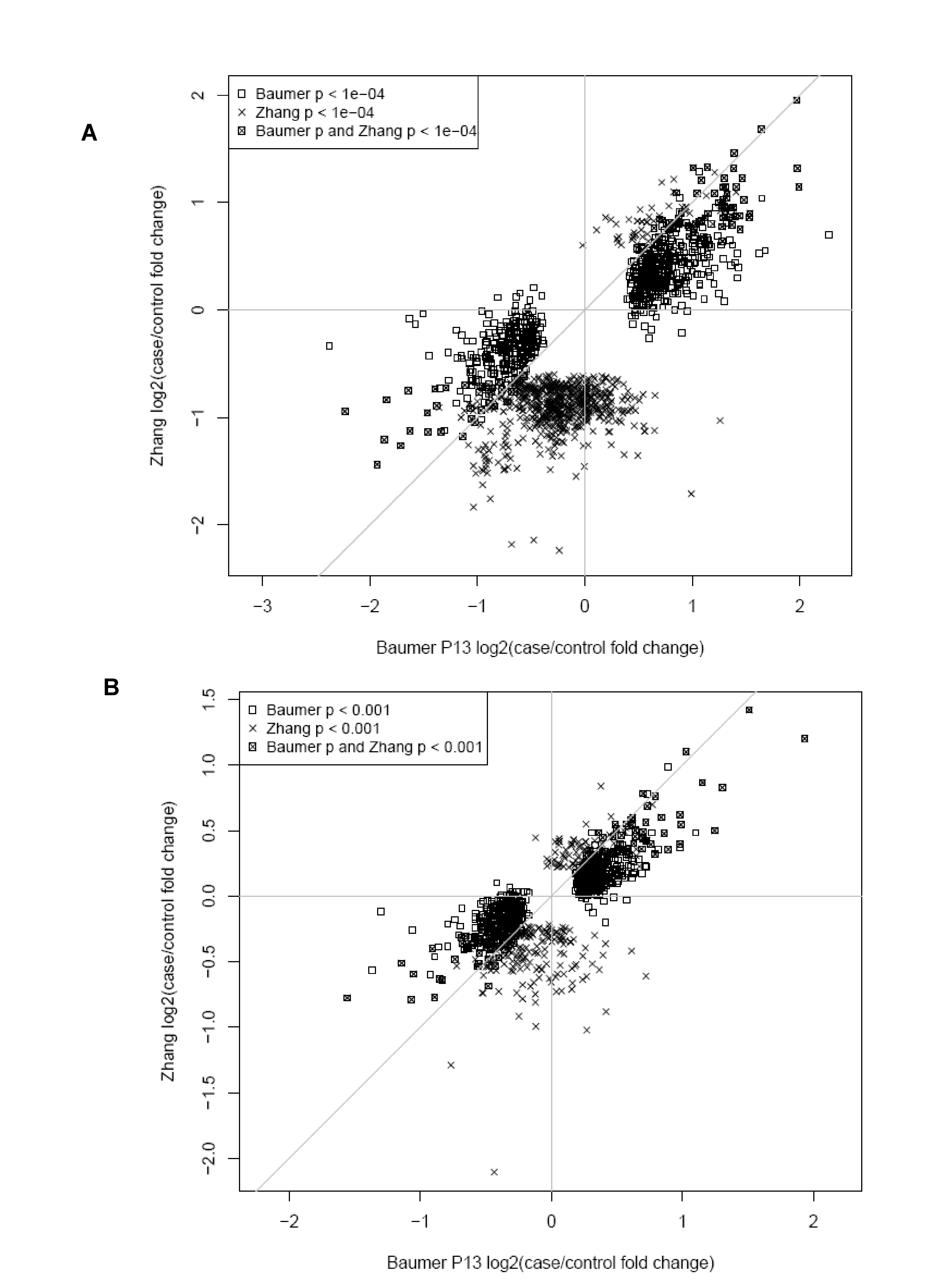

Supplement: Figure S7 — Comparison of P13 data set and Zhang et al data set. This figure (ENSE (A), ENSG (B)) compares the log2(case/control fold change) across studies. Only probe sets that are significantly differentially expressed in at least one study are included. There is clearly a degree of concordance between the two studies at these probe sets. In particular, the directionality of differential expression is extremely consistent across studies (Table S2). (0.63 MB TIF) [file pgen.1000773.s007.tif]
