# Supplementary material for: Alternative Splicing Events Are a Late Feature of Pathology in a Mouse Model of Spinal Muscular Atrophy
Source: PLoS Genet. 2009 Dec 18;5(12):e1000773. doi: 10.1371/journal.pgen.1000773 (PMC2787017; doi:10.1371/journal.pgen.1000773)
Supplement: Table S1 — P13 gene level changes SMA vs control, fold change >1.5, P≤0.05. (0.22 MB DOC) [file pgen.1000773.s008.doc]

| **Transcripts Cluster Id** | **Fold change** | **Regulation** | **Gene Title** | **Gene Symbol** |
| --- | --- | --- | --- | --- |
| 6849595 | 3.9 | up | cyclin-dependent kinase inhibitor 1A (P21) | Cdkn1a |
| 6822477 | 3.5 | up | cDNA sequence BC055107 | BC055107 |
| 6842587 | 3.5 | down | Chondrolectin | Chodl |
| 6805270 | 3.1 | down | --- | --- |
| 6971280 | 3.1 | up | sulfotransferase family 1A, phenol-preferring, member 1 | Sult1a1 |
| 7014503 | 2.9 | down | aminolevulinic acid synthase 2, erythroid | Alas2 |
| 6809524 | 2.9 | down | survival motor neuron 1 | Smn1 |
| 6969997 | 2.7 | down | hemoglobin, beta adult minor chain /// hemoglobin, beta adult major chain | Hbb-b2 /// Hbb-b1 |
| 6854604 | 2.6 | up | FK506 binding protein 5 | Fkbp5 |
| 6784062 | 2.5 | down | insulin-like growth factor binding protein 4 | Igfbp4 |
| 6899760 | 2.5 | up | thioredoxin interacting protein | Txnip |
| 6874653 | 2.4 | up | coiled-coil domain containing 3 | Ccdc3 |
| 6802026 | 2.3 | up | pleckstrin 2 | Plek2 |
| 6766301 | 2.2 | up | PERP, TP53 apoptosis effector | Perp |
| 6785865 | 2.2 | up | insulin-like growth factor binding protein 3 | Igfbp3 |
| 6893002 | 2.1 | down | solute carrier family 13 (sodium-dependent dicarboxylate transporter), member 3 | Slc13a3 |
| 6783673 | 2.1 | up | Chondroadherin | Chad |
| 6837006 | 2.1 | down | Parvalbumin | Pvalb |
| 6973586 | 2.1 | up | apolipoprotein C-I /// golgi coiled coil 1 | Apoc1 /// Gcc1 |
| 6854401 | 2.1 | down | hematological and neurological expressed 1-like | Hn1l |
| 6947932 | 2.0 | up | Kruppel-like factor 15 | Klf15 |
| 6755054 | 2.0 | down | regulator of G-protein signaling 5 | Rgs5 |
| 6825445 | 2.0 | up | PTK2 protein tyrosine kinase 2 beta | Ptk2b |
| 6941029 | 2.0 | up | peroxisomal membrane protein 2 | Pxmp2 |
| 6986064 | 2.0 | up | angiotensinogen (serpin peptidase inhibitor, clade A, member 8) | Agt |
| 6965670 | 2.0 | up | hypoxia inducible factor 3, alpha subunit | Hif3a |
| 6766455 | 2.0 | up | serum/glucocorticoid regulated kinase 1 | Sgk1 |
| 6755055 | 1.9 | down | regulator of G-protein signaling 5 | Rgs5 |
| 6792813 | 1.9 | up | dicarbonyl L-xylulose reductase | Dcxr |
| 6805381 | 1.9 | up | histone cluster 1, H1c | Hist1h1c |
| 7009834 | 1.9 | down | solute carrier family 38, member 5 | Slc38a5 |
| 7016678 | 1.9 | down | Apelin | Apln |
| 6791298 | 1.9 | down | topoisomerase (DNA) II alpha | Top2a |
| 6901353 | 1.9 | up | alanine-glyoxylate aminotransferase 2-like 1 | Agxt2l1 |
| 6850019 | 1.9 | up | transporter 1, ATP-binding cassette, sub-family B (MDR/TAP) | Tap1 |
| 6769197 | 1.9 | up | cold inducible RNA binding protein | Cirbp |
| 6805380 | 1.8 | down | histone cluster 1, H2bb /// histone cluster 1, H2be /// histone cluster 1, H2bg /// predicted gene, OTTMUSG00000013203 /// histone cluster 1, H2bc | Hist1h2bb /// Hist1h2be /// Hist1h2bg /// RP23-38E20.1 /// Hist1h2bc |
| 7009748 | 1.8 | down | diacylglycerol kinase kappa | Dgkk |
| 6750440 | 1.8 | down | insulin-like growth factor binding protein 2 | Igfbp2 |
| 6781090 | 1.8 | up | glutathione peroxidase 3 | Gpx3 |
| 6774264 | 1.8 | up | DNA-damage-inducible transcript 4 | Ddit4 |
| 6939671 | 1.8 | down | transmembrane protease, serine 11d | Tmprss11d |
| 6987513 | 1.8 | up | neuropeptide S receptor 1 | Npsr1 |
| 6978290 | 1.8 | up | metallothionein 2 | Mt2 |
| 6789367 | 1.8 | up | solute carrier family 2 (facilitated glucose transporter), member 4 | Slc2a4 |
| 6819275 | 1.8 | up | leukotriene B4 receptor 2 | Ltb4r2 |
| 6753068 | 1.8 | down | RIKEN cDNA 5430435G22 gene | 5430435G22Rik |
| 6891880 | 1.8 | down | CD93 antigen | Cd93 |
| 6783997 | 1.7 | up | protein phosphatase 1, regulatory (inhibitor) subunit 1B | Ppp1r1b |
| 6892899 | 1.7 | down | secretory leukocyte peptidase inhibitor | Slpi |
| 6820325 | 1.7 | down | lymphocyte cytosolic protein 1 | Lcp1 |
| 6787918 | 1.7 | down | proteasome (prosome, macropain) 28 subunit, beta /// protease (prosome, macropain) 28 subunit beta B, pseudogene | Psme2 /// Psme2b-ps |
| 6990715 | 1.7 | up | CD109 antigen | Cd109 |
| 6961201 | 1.7 | up | small nuclear ribonucleoprotein polypeptide A' | Snrpa1 |
| 6844316 | 1.7 | up | proline dehydrogenase | Prodh |
| 6889357 | 1.7 | up | proline rich Gla (G-carboxyglutamic acid) 4 (transmembrane) | Prrg4 |
| 6781984 | 1.7 | up | period homolog 1 (Drosophila) | Per1 |
| 6863755 | 1.7 | up | desmocollin 3 | Dsc3 |
| 6978291 | 1.7 | up | metallothionein 1 | Mt1 |
| 6785114 | 1.7 | down | RAB37, member of RAS oncogene family | Rab37 |
| 6985813 | 1.7 | down | potassium voltage-gated channel, subfamily G, member 4 | Kcng4 |
| 6767782 | 1.7 | up | glycoprotein 49 A /// leukocyte immunoglobulin-like receptor, subfamily B, member 4 | Gp49a /// Lilrb4 |
| 6972205 | 1.7 | up | leucine-rich and death domain containing | Lrdd |
| 6994935 | 1.7 | down | sterol-C5-desaturase (fungal ERG3, delta-5-desaturase) homolog (S. cerevisae) | Sc5d |
| 6885616 | 1.7 | up | RIKEN cDNA 1700007K13 gene | 1700007K13Rik |
| 6961987 | 1.7 | down | protein regulator of cytokinesis 1 | Prc1 |
| 6770072 | 1.7 | down | Lumican | Lum |
| 6966610 | 1.7 | up | pleckstrin homology domain containing, family F (with FYVE domain) member 1 | Plekhf1 |
| 6949591 | 1.7 | up | solute carrier family 6 (neurotransmitter transporter, GABA), member 13 | Slc6a13 |
| 6772802 | 1.7 | down | ectonucleotide pyrophosphatase/phosphodiesterase 1 | Enpp1 |
| 6813317 | 1.7 | up | ribosomal protein S24 | Rps24 |
| 6861662 | 1.7 | up | phorbol-12-myristate-13-acetate-induced protein 1 | Pmaip1 |
| 6823666 | 1.6 | up | protein kinase C, delta | Prkcd |
| 6926165 | 1.6 | up | complement component 1, q subcomponent, beta polypeptide | C1qb |
| 6854487 | 1.6 | up | dual specificity phosphatase 1 | Dusp1 |
| 7013389 | 1.6 | down | kelch-like 4 (Drosophila) | Klhl4 |
| 6939241 | 1.6 | down | kinase insert domain protein receptor | Kdr |
| 6988366 | 1.6 | up | sodium channel, voltage-gated, type III, beta | Scn3b |
| 6762345 | 1.6 | up | B-cell translocation gene 2, anti-proliferative | Btg2 |
| 6810066 | 1.6 | down | DEP domain containing 1B | Depdc1b |
| 6803284 | 1.6 | up | serine (or cysteine) peptidase inhibitor, clade A, member 3C | Serpina3c |
| 6878448 | 1.6 | down | integrin alpha 4 /// ceramide kinase-like | Itga4 /// Cerkl |
| 6929660 | 1.6 | down | abhydrolase domain containing 1 | Abhd1 |
| 6978336 | 1.6 | up | chemokine (C-C motif) ligand 17 | Ccl17 |
| 6890120 | 1.6 | down | G protein-coupled receptor 176 | Gpr176 |
| 6882333 | 1.6 | down | TPX2, microtubule-associated protein homolog (Xenopus laevis) | Tpx2 |
| 6789540 | 1.6 | down | smoothelin-like 2 | Smtnl2 |
| 6792887 | 1.6 | up | zinc finger protein 750 | Zfp750 |
| 6866653 | 1.6 | up | mitogen-activated protein kinase 4 | Mapk4 |
| 6788655 | 1.6 | down | gap junction protein, gamma 2 | Gjc2 |
| 6768123 | 1.6 | down | procollagen-proline, 2-oxoglutarate 4-dioxygenase (proline 4-hydroxylase), alpha 1 polypeptide | P4ha1 |
| 6911679 | 1.6 | up | transformation related protein 53 inducible nuclear protein 1 | Trp53inp1 |
| 7012866 | 1.6 | down | gap junction protein, beta 1 | Gjb1 |
| 6876052 | 1.6 | up | phytanoyl-CoA dioxygenase domain containing 1 /// leucine rich repeat containing 8A | Phyhd1 /// Lrrc8a |
| 6929861 | 1.6 | up | regulator of G-protein signaling 12 | Rgs12 |
| 6936723 | 1.6 | up | ATG9 autophagy related 9 homolog B (S. cerevisiae) | Atg9b |
| 6943797 | 1.6 | down | guanine nucleotide binding protein (G protein), gamma 11 | Gng11 |
| 6796902 | 1.6 | up | thyroid stimulating hormone receptor | Tshr |
| 6864680 | 1.6 | down | heparin-binding EGF-like growth factor | Hbegf |
| 6762944 | 1.6 | down | phospholipase A2, group IVA (cytosolic, calcium-dependent) | Pla2g4a |
| 6990327 | 1.6 | up | aldehyde dehydrogenase family 1, subfamily A2 | Aldh1a2 |
| 6813246 | 1.6 | up | nuclear factor, interleukin 3, regulated | Nfil3 |
| 6956912 | 1.6 | up | adiponectin receptor 2 | Adipor2 |
| 6837375 | 1.6 | up | cytochrome P450, family 2, subfamily d, polypeptide 22 | Cyp2d22 |
| 6963623 | 1.6 | down | calcitonin-related polypeptide, beta | Calcb |
| 6865221 | 1.6 | down | tripartite motif-containing 36 | Trim36 |
| 6835353 | 1.6 | up | angiopoietin 1 | Angpt1 |
| 6854304 | 1.6 | up | spermine binding protein-like /// spermine binding protein | Sbpl /// Sbp |
| 6881087 | 1.6 | up | c-mer proto-oncogene tyrosine kinase | Mertk |
| 6962930 | 1.6 | down | procollagen-proline, 2-oxoglutarate 4-dioxygenase (proline 4-hydroxylase), alpha polypeptide III | P4ha3 |
| 6852882 | 1.6 | up | tumor-associated calcium signal transducer 1 | Tacstd1 |
| 6933591 | 1.6 | up | D-amino acid oxidase 1 | Dao1 |
| 6817645 | 1.6 | up | double homeobox B-like /// predicted gene, ENSMUSG00000072675 /// predicted gene, ENSMUSG00000072672 | Duxbl /// ENSMUSG00000072675 /// ENSMUSG00000072672 |
| 6819883 | 1.5 | down | PDZ binding kinase | Pbk |
| 6849551 | 1.5 | up | RIKEN cDNA E230001N04 gene | E230001N04Rik |
| 7016340 | 1.5 | down | NADH dehydrogenase (ubiquinone) 1 alpha subcomplex, 1 | Ndufa1 |
| 6926166 | 1.5 | up | complement component 1, q subcomponent, C chain | C1qc |
| 6933812 | 1.5 | down | Tescalcin | Tesc |
| 6987954 | 1.5 | down | E26 avian leukemia oncogene 1, 5' domain | Ets1 |
| 6892852 | 1.5 | up | adenosine deaminase | Ada |
| 6754149 | 1.5 | up | glutamate-ammonia ligase (glutamine synthetase) | Glul |
| 6791257 | 1.5 | down | RIKEN cDNA 1810046J19 gene | 1810046J19Rik |
| 6963131 | 1.5 | up | olfactory receptor 646 | Olfr646 |
| 6756790 | 1.5 | up | myeloblastosis oncogene-like 1 | Mybl1 |
| 6971848 | 1.5 | down | antigen identified by monoclonal antibody Ki 67 | Mki67 |
| 6815291 | 1.5 | up | polymerase (DNA directed), kappa | Polk |
| 6985850 | 1.5 | up | RIKEN cDNA 1190005I06 gene | 1190005I06Rik |
| 7023132 | 1.5 | up | RIKEN cDNA D030013I16 gene /// phosphatidylserine decarboxylase pseudogene /// RIKEN cDNA 4933439C20 gene | D030013I16Rik /// LOC236604 /// 4933439C20Rik |
| 6939270 | 1.5 | down | neuromedin U | Nmu |
| 6943310 | 1.5 | down | heat shock 105kDa/110kDa protein 1 | Hsph1 |
| 6949853 | 1.5 | down | mitochondrial ribosomal protein L51 | Mrpl51 |
| 6824940 | 1.5 | up | gap junction protein, beta 6 | Gjb6 |
| 6961912 | 1.5 | down | Aggrecan | Acan |
| 6957240 | 1.5 | up | potassium voltage-gated channel, shaker-related subfamily, member 5 | Kcna5 |
| 6785399 | 1.5 | up | brain-specific angiogenesis inhibitor 1-associated protein 2 | Baiap2 |
| 6949310 | 1.5 | up | RIKEN cDNA 8430408G22 gene | 8430408G22Rik |
| 6758435 | 1.5 | down | solute carrier family 40 (iron-regulated transporter), member 1 | Slc40a1 |
| 6903558 | 1.5 | up | carboxypeptidase B1 (tissue) | Cpb1 |
| 6925362 | 1.5 | down | eukaryotic translation initiation factor 2C, 3 | Eif2c3 |
| 6836700 | 1.5 | up | lymphocyte antigen 6 complex, locus D | Ly6d |
| 6976237 | 1.5 | down | hydroxyprostaglandin dehydrogenase 15 (NAD) | Hpgd |
| 6854760 | 1.5 | down | glyoxalase 1 | Glo1 |

**Table S1:** P13 gene level changes SMA vs control, fold change >1.5, P≤0.05
