# Supplementary material for: Alternative Splicing Events Are a Late Feature of Pathology in a Mouse Model of Spinal Muscular Atrophy
Source: PLoS Genet. 2009 Dec 18;5(12):e1000773. doi: 10.1371/journal.pgen.1000773 (PMC2787017; doi:10.1371/journal.pgen.1000773)
Supplement: Text S1 — Statistical analysis of microarray findings; comparison of the P13 dataset with Zhang et al; primer sequences. (0.09 MB PDF) [file pgen.1000773.s018.pdf]

Statistical analysis of microarray findings

Twenty-four CEL files (four control mice plus four SMA mice, at each of three time points) were preprocessed concurrently using RMA without background correction (i.e. quantile normalization followed by robust probe-set summarization). RMA background correction was omitted because it had an empirically undesirable effect on the data, as is now described. RMA background applies a smooth, monotonic transformation from raw probe intensities to corrected probe intensities (see Figure S4 for the data-based mapping for a single array). The function is linear for medium-to-high intensities, but tends to stretch out the low-intensity range (the figure is annotated with a two-fold interval that is mapped to a ten-fold interval). It does not make sense to inflate (relatively) that variation in intensity which is near to the background detection level. This is because such variation is relatively unlikely to be representative of true signal, and likely to represent experimental noise.

Publicly available custom chip-definition files (CDFs) were downloaded from http://brainarray.mbni.med.umich.edu/Brainarray/Database/CustomCDF/CDF_download.asp and used to group probes into sets. Parallel analyses, based on two different CDFs were performed. The first CDF, which we refer to as ENSE, defines a probe set for each Ensembl exon. The second, ENSG, defines a probe set for each Ensembl gene. See [27] for a description of how these CDFs are created and a comparison of their properties with those of Affymetrix's annotation system.

There were 211,567 and 211,911 probe sets for the ENSE and ENSG analyses respectively. Each preprocessed data set comprises a single summary for each probe set on each array, representing the $\log_2$(expression) at the corresponding probe set in the assayed sample.

At each probe set, the following linear model was fit using the limma package, version 2.16.5 [29,47]

$$y_i = \alpha_{t(i)} + s(i)\beta + g(i)\gamma_{t(i)} + \varepsilon_i \ ,$$

where $i \epsilon \{1, \ldots, 24\}$ denotes subject number; $s(i)$ indicates whether i is male $(s = 0)$ or female $(s = 1)$; $t(i) \in \{P1, P7, P13\}$ denotes the time of sampling of the ith subject; $g(i)$ indicates whether subject i is wild type $(g = 0)$ or SMN deficient $(g = 1)$; ε_i denotes the residual error, which is assumed (across subjects) to be independently Gaussian distributed with zero mean and common variance. The fitted model comprises estimates of seven parameters, $(\alpha_{P1}, \alpha_{P7}, \alpha_{P13}, \beta, \gamma_{P1}, \gamma_{P7}, \gamma_{P13})$, of which $\gamma_{P1}, \gamma_{P7}, \gamma_{P13}$ are of primary interest, representing case/control differences within time point. For each γ_t, a p-value was calculated for the t-test of the null hypothesis $\gamma_t = 0$ against the two-sided alternative. Let p_{jt} denote the p-value at the jth probe set, for the test of $\gamma_t = 0,\ t \in \{P1, P7, P13\}$.

To assess the number of false positives, a permutation-based analysis was conducted. Each permutation of the data retained the time-point labels and sex labels of each subject, but randomly permuted the case/control labels of the subjects within each time point (i.e. for each time point, four of the eight assays were randomly labelled as cases, and four as controls). The data were permuted in this way 200 times. Each permuted data set was statistically analysed in the same way as the actual data set, giving three p-values (one for each time point) for each probe set. Let $p_{jt}^{(\pi)}$ denote the p-value at the πth permutation, at the jth probe set, for the test of $\gamma_t = 0$.

For a particular p-value cut-off, λ say, the number of significant probe sets at time point t is $|\{j : j = 1, \dots, P,\ p_{jt} < \lambda\}|$, where P denotes the total number of probe sets (in ENSE or ENSG). The number of false-positive probe sets at time point t is estimated by the median of the set $\left\{\left|\left\{j : j = 1, \dots, P,\ p_{jt}^{(\pi)} < \lambda\right\}\right| : \pi = 1, \dots, 200\right\}$, and the false discovery rate (FDR) is estimated in the obvious way as the ratio: (estimated number of false positives) / (number of significant probe sets for the actual data set). Table S1 displays the number of significant results at each time point, along with the estimated FDR, for a number of different choices of p-value cut-off, λ. Subsequent results are presented with $\lambda = 10^{-4}$ for ENSE, and with $\lambda = 10^{-3}$ for ENSG, as these choices control the FDR at a reasonably low level. For ENSE there are 72 (0.35), 66 (0.06), and 812 (0.01) significant exons at P1, P7 and P13 respectively (estimated FDR in parentheses). For ENSG, there are 83 (0.23), 92 (0.09), and 693 (0.02) significant genes at P1, P7 and P13 respectively. Figures 4B and S1 display the sharing of significant probe sets across time points. A consistent qualitative picture emerges across the two preprocessed data sets: case/control differential expression is much more widespread at P13 than at either of the earlier time points.

While it is reasonable to compare across time points within ENSE or ENSG, a direct comparison between ENSE and ENSG (of the number of significant probe sets) is ill advised, as the two analyses have different power to detect differential expression. The ENSG analysis has more power to detect whole-transcript differential expression than does ENSE (more probes in a probe set increases the signal-to-noise ratio). However, ENSE has more power than ENSG to detect differential

splicing that manifests itself as differences at one or a few exons (with ENSE having the biggest advantage over ENSG for single exon-skipping events).

Statistical analyses were performed using R [28], version 2.8.1.

Comparison of the P13 dataset with Zhang et al.

In order to allow direct comparison, we preprocessed Zhang *et al.*'s data [15] in an identical fashion to the data in our study (all six arrays preprocessed together using RMA without background correction, employing two parallel probe-set groupings: ENSE and ENSG). A linear model with terms for sex and SMA/control status was fitted at each probe set [29,49]. The same p-value thresholds were applied to both the P13 data and Zhang data (1e-4 for ENSE and 1e-3 for ENSG). In the ENSE analysis, Zhang's data yielded 768 significantly differentially expressed exons, of which 94 are represented amongst the 812 significant exons our P13 data (this is a higher level of sharing than would be expected to occur by chance: p < 1e-107). In the ENSG analysis Zhang yields 382 genes, our data 693, with an overlap of 119 (again this overlap is unlikely to have occurred by chance: p < 1e-84).

The directionality of differential expression is extremely consistent across studies, as is demonstrated in Table S2 (+ = cases over-expressed relative to controls).

Primer sequences

qRT-PCR

**SYBR Green qPCR**	**Forward primer 5'-3'**	**Reverse primer 5'-3'**
mGAPDH	TGTGTCCGTCGTGGATCTGA	CCTGCTTCACCACCTTCTTGA
Chodl 1-2	GGTCAGTGGTCAAAAGGTGTGTT	AAGCTCACCCGGCTAGACAGT
Chodl-001 5/6	TGCTTTGGGAACCTGCTGTT	CGGGCTAGTTTTTGATCTTCCTT
Chodl-002 5/6	GTTTCCAGATGTTGCATAAAAGGA	AGATGATAATGGAGTGGAGTCTTTGA
Snrpa1	TGACAATGAGATCCGGAAACTG	CACCTATACGGCAAATTCTGTTGTT
Mccc2	CAGAGCATACAGTCCAAGGTTTCTC	GCCTGCTCTCCTCCCATCA
Cdkn1	GGCAGACCAGCCTGACAGAT	TTCAGGGTTTTCTCTTGCAGAAG
Uspl1-2	AGAGGAGTTCGGGTCCACTGT	CAATGAACCCCTCCCGAAGT
Uspl1 9-10	CTTCCATGCATGAAGCCAAA	GGCAGCAACTGTGTCTGAGAGT
ChAT	AATGGCGTCCAACGAGGAT	CGGTTGGTGGAGTCTTTTAAGAG

Semi-quantitative RT-PCR

	**Forward primer 5'-3'**	**Reverse primer 5'-3'**
Uspl1 1-3	CTGTATGCGAAGAAGGAGCG	TTTCGCTCTACAAGCAGGGC
Mccc2-201	CAGGAGCTGCAGAACCGAGC	GGGTTACATCCTGAAGATGCC
Mccc2-203	GAGGTGAAGGCAGCTACAGG	AGCGGTAAGATGGGACATCC
